# α6GABA_A_ receptor-selective positive allosteric modulator as a novel therapy for fibromyalgia: A proof-of-concept study in mice modeling chronic widespread musculoskeletal pain

**DOI:** 10.1016/j.neurot.2026.e00956

**Published:** 2026-07-16

**Authors:** Myles Sant-Cassia, Cheng-Han Lee, V.V.N. Phani Babu Tiruveedhula, James Cook, Chih-Cheng Chen, Lih-Chu Chiou

**Affiliations:** a Chemical Biology and Molecular Biophysics Program, Taiwan International Graduate Program, Academia Sinica, Taipei 115201, Taiwan; b Department and Graduate Institute of Pharmacology, College of Medicine, National Taiwan University, Taipei 100233, Taiwan; c Institute of Biomedical Sciences, Academia Sinica, Taipei 115201, Taiwan; d Department of Chemistry and Biochemistry, Milwaukee Institute for Drug Discovery, University of Wisconsin-Milwaukee, Milwaukee, Wisconsin, WI, 53211, USA; e Graduate Institute of Biochemical Sciences, National Taiwan University, Taipei 106319, Taiwan; f Graduate Institute of Brain and Mind Sciences, College of Medicine, National Taiwan University, Taipei 100233, Taiwan; g Graduate Institute of Acupuncture Science, China Medical University, Taichung 404802, Taiwan

**Keywords:** Fibromyalgia, GABA_A_ receptor, Positive allosteric modulator, Nociplastic pain, Spinal cord, Dorsal root ganglia

## Abstract

Fibromyalgia, a chronic pain disorder mainly due to central sensitization, remains an unmet medical need, as available treatments provide limited efficacy and are frequently associated with dose-limiting adverse effects. We previously developed pyrazoloquinolinone (PQ) compounds as the first positive allosteric modulators that selectively bind to GABA_A_ receptors containing the α6 subunit (α6GABA_A_Rs), among others. Here, we found that PQ Compound 6 and its deuterated, druggable candidate, DK-I-56-1, alleviated mechanical allodynia and thermal hyperalgesia in fibromyalgia models induced by dual acidic-saline injections and intermittent cold stress in ICR mice. The antinociceptive effects of PQs were sexually dimorphic, with greater sensitivity in females, and did not develop tolerance after repeated administrations. Moreover, their antinociceptive effects were prevented by furosemide, an α6GABA_A_R antagonist, administered by intrathecal, but not systemic, injection, and were abolished in *Gabra6* (the α6 subunit-encoding gene)-knockout mice, suggesting the involvement of spinal α6GABA_A_Rs. Immunofluorescent staining supports the presence of α6GABA_A_Rs in the spinal dorsal horn and co-localization with NeuN. DK-I-56-1 produced anti-nociceptive efficacy (10 mg/kg in males and 3 mg/kg in females) comparable to gabapentin (10 mg/kg) following *i.p.* administration, and exhibited an additive effect with gabapentin when co-administered at low doses. These results suggest that the α6GABA_A_R-selective positive allosteric modulator, such as DK-I-56-1, is a potential strategy for fibromyalgia pain management as monotherapy or as adjuncts to gabapentinoids.

## Graphical abstract

**Figure:**
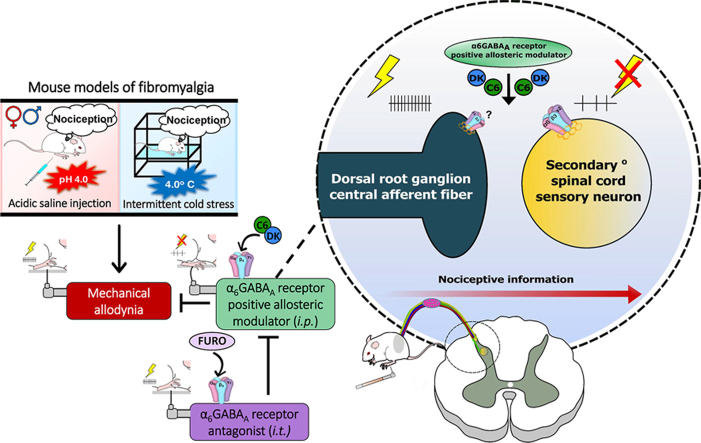

## Introduction

Fibromyalgia is a chronic nociplastic pain disorder characterized by widespread musculoskeletal pain, fatigue, mood disturbance, and cognitive deficits [1]. Affecting 4–8% of people (predominantly females), it is the third most common cause of musculoskeletal pain [2,3]. While a cure for fibromyalgia remains lacking, gabapentinoids and antidepressants may treat pain symptoms in patients with fibromyalgia. However, their usage is limited by low response rates, modest efficacy, and intolerable side effects [[4], [5], [6], [7]]. Thus, a paramount need remains to develop new pharmacotherapies for fibromyalgia.

While peripheral nociceptive inputs and small-fiber abnormalities have been implicated in chronic widespread pain, these factors are insufficient to fully account for the central amplification and persistent characteristic of fibromyalgia [8]. Thus, it is considered a central nervous system disorder, with central sensitization (including spinal and supraspinal sensitization) [9] as the central hypothesis of fibromyalgia. This may be attributed to an impaired excitatory–inhibitory balance in the pain regulatory pathways. GABAergic inhibitory circuits play a key role in maintaining the excitatory–inhibitory imbalance in these pathways, and their dysfunction can underlie the chronic nociplastic pain characteristic of fibromyalgia.

In rodents, GABA has been shown to be antinociceptive [10]. Reduced GABA release or GABA_A_ receptor (GABA_A_R) activation contributes to central sensitization by reducing disinhibition, and contributing to synaptic facilitation, leading to enlarged neuron receptive fields, and increased spontaneous neuronal activity [11,12]. Clinically, fibromyalgia patients may have lower GABA levels in the cerebrospinal fluid [13], and a higher binding potential of ^18^[F]flumazenil (a GABA_A_R PET ligand) in the whole brain [14], suggesting an upregulation of GABA_A_Rs. Furthermore, a single nucleotide polymorphism of the gene encoding the β3 subunit of GABA_A_R, *GABRB3*, was reported in fibromyalgia patients [15].

GABA_A_Rs are pentameric chloride channels, typically consisting of two α, two β and one γ or δ subunits [16]. Among others, the GABA_A_Rs containing the *Gabra6* gene-encoded α6 subunit (α6GABA_A_Rs) are noteworthy. α6GABA_A_Rs are densely and almost exclusively expressed in cerebellar granule cells (GCs) throughout the brain [17], while also present, but less abundantly, in the spinal dorsal horn [17,18] and dorsal root ganglia (DRG) [19], key pain processing areas implicated in the regulation of nociplastic pain, including fibromyalgia. As high-affinity GABA_A_Rs, α6GABA_A_Rs function to mediate tonic inhibition driven by ambient released GABA [20]. Thus, α6GABA_A_Rs are well-positioned to regulate nociception signalling in disease states characterized with reduced GABAergic tone, such as fibromyalgia [13,21].

In addition to their limited distribution in the brain, α6GABA_A_Rs have been understudied due to a lack of selective ligands. Previously, we identified several pyrazoloquinolinones (PQs) as α6GABA_A_R-selective positive allosteric modulators (PAMs) [22]. They displayed micromolar binding affinity at the α+β-interface, distinct from the binding sites of GABA and benzodiazepines (BDZs) at α-β+ and α+γ-interfaces, respectively, on GABA_A_Rs. Using PQ Compound 6 and its deuterated derivative, DK-I-56-1, our groups and others have demonstrated that α6GABA_A_R-PAMs exert robust antinociceptive effects across multiple rodent models of chronic pain, including migraine [[23], [24], [25]], trigeminal neuropathic pain [26], and spinal nerve injury-induced neuropathic pain [19]. In this study, we further investigated whether PQs could relieve pain in fibromyalgia.

Clinical manifestations of chronic musculoskeletal pain in fibromyalgia can be mimicked in mice with dual acidic-saline injections (ASI), which induces long-lasting mechanical allodynia, but not thermal hyperalgesia, due to central sensitization by a peripheral insult [27]. Additionally, intermittent cold stress (ICS) can produce long-term mechanical allodynia and thermal hyperalgesia due to chronic cold stress-induced central sensitization without tissue injury, representing nociplastic pain in patients with fibromyalgia [28]. Here, we employed these two fibromyalgia models by assessing ASI-induced mechanical allodynia and ICS-induced mechanical allodynia and thermal hyperalgesia in both sexes of mice treated with Compound 6 or DK-I-56-1. To elucidate the contribution of α6GABA_A_Rs in effects of PQs and their site of action, we utilized mouse genetic and pharmacological approaches, respectively, using *Gabra6* global knockout (*Gabra6*-KO) mice, and the pharmacological blockade of α6GABA_A_Rs by furosemide, an α6GABA_A_R-selective antagonist [29], by systemic or intrathecal administration. Finally, we also benchmarked the therapeutic effects of PQs against gabapentin, a clinically used fibromyalgia medication [30].

## Materials and Methods

### Animals

All animal experiments were approved by the Institutional Animal Care and Use Committees (IACUC) of National Taiwan University, College of Medicine, Taipei, Taiwan (Project No. 20230413) and Academia Sinica, Taipei, Taiwan (Project No. 24122346), respectively. Adult male and female (6–9 weeks old, 24–34 g) ICR mice purchased from Lesco Biotechnology Co., Ltd (Bio LASCO) were housed 4- to 5-per cage in a holding room with a 12:12 light-dark cycle at 22 ± 2 °C, with food and water *ad libitum*. Behavioral experiments were conducted during the light cycle. On the day of experiments, mice with their cages were moved to the behavioral room and acclimated there for at least 1 h before experiments. The experimenter was blind to the treatments.

#### *Gabra6*-KO mice

We generated *Gabra6*-KO mice using the ICR strain, which were employed in all of our PQ studies previously, by using CRISPR/Cas genome editing, to remove the *Gabra6* gene (Transcript ID: ENSMUST00000155218.9, the Mouse Genome Informatics database) [31]. Two single-guide RNAs (sgRNA), 5′-TTATATACACAAAGCTGTGC-3' (5′ sgRNA) and 5′-TCTCTATCTTCATCTAAGCT-3′ (3′ sgRNA), were designed to target the exon 1 upstream (0.9 kb) and exon 9 downstream (0.7 kb) of *Gabra6*, respectively. This large-scale genomic region deletion, a ∼14.3-kb deletion encompassing exons 1–9 of the *Gabra6* locus, was used to help prevent potential alternative splicing, exon skipping, or alternative translation initiation. The Cas9 protein and sgRNAs were introduced into fertilized embryos by electroporation (see Supplementary Methods). Founder mice carrying the intended deletion were identified by PCR genotyping, and subsequently bred to establish the *Gabra6* knockout colony. Three heterozygous parents were used to breed the offspring with three genotypes: homozygous (*Gabra6* ^*−/−*^*),* heterozygous (*Gabra6* ^*+/−*^), and wild-type (*Gabra6* ^*+/+*^). Littermates were backcrossed with more than four generations before being used. Genotyping was performed with the tail tissue by the polymerase chain reaction (PCR) using four primers: *Gabra6*-5′ forward (5′-ACGTCTCTCATATGTGCTGTGTT-3′), *Gabra6*-5′ reverse (5′-ACAATTTCCTTCTTTTGGACAGCA-3′), *Gabra6*-3′ forward (5′-TGGACTGAATCTCAGAATCCATTT-3′), and *Gabra6*-3′ reverse (5′-AGCTGTAGTACATTAATGGGTTGTG-3′) (Supplementary Fig. s1a).

### Measuring nociceptive responses

#### Mechanical allodynic responses

Hind paw mechanosensitivity was measured using the Simplified Up-Down (SUDO) method [32]. Mice were placed individually within clear Perspex containers (10∗6∗10∗ cm^3^) above a steel mesh grid floor (30 cm in height), and allowed to habituate 1-h before testing. A total of five von Frey (vF) monofilament fibers (Aesthesio) were used, starting at 0.40 g, and ranging from 0.02 g to 2.0 g. Each fiber was applied perpendicular to the hind paw plantar surface for 5 s to measure the paw withdrawal threshold (PWT) of the tested mouse. Vocalization or lifting and licking of the targeted paw was considered a positive nociceptive response. The baseline PWT in each mouse was recorded before the ASI or ICS induction. Mice with a PWT lower than the baseline PWT, were considered to have mechanical allodynia. The cut-off von-Frey filament was set at 2.0 g due to the force of stronger filaments lifting the mice hind paw without the filament buckling.

#### Thermal hyperalgesia responses

Thermal paw withdrawal latency (PWL) was assessed using the Hargreaves method (IITC Plantar Test Apparatus, IITC Inc. Life Science) [33]. Mice were individually placed within clear Perspex compartments (11 cm × 20 cm × 15 cm per compartment), placed on top of the Hargreaves glass panel. A focused beam of radiant light (40% active intensity) was applied perpendicular to the hind paw plantar surface, with a 20-s cut-off time to avoid tissue damage. The latency to paw withdrawal or licking (PWL) was recorded. The stimulus was repeated three times on each hind paw, with an interval of at least 5-min.

### Fibromyalgia models

#### The ASI model

The ASI model was induced by dual acidic-saline injections in ICR mice as previously described [27], with modifications. On day 0, following baseline mechanical PWT assessment, mice were anesthetized by 2% isoflurane. Then the first acidic-saline injection was performed by intramuscular injection of 20 μL 2-(N-morpholino)ethanesulfonic acid-buffered pH4.0 saline into the left gastrocnemius muscle. A second acidic-saline injection was conducted after 24 h. The control group received 4-(2-hydroxyethyl)-1-piperazineethanesulfonic acid-buffered (pH7.4) saline injections twice, following the same protocol.

**Fig. 1:**
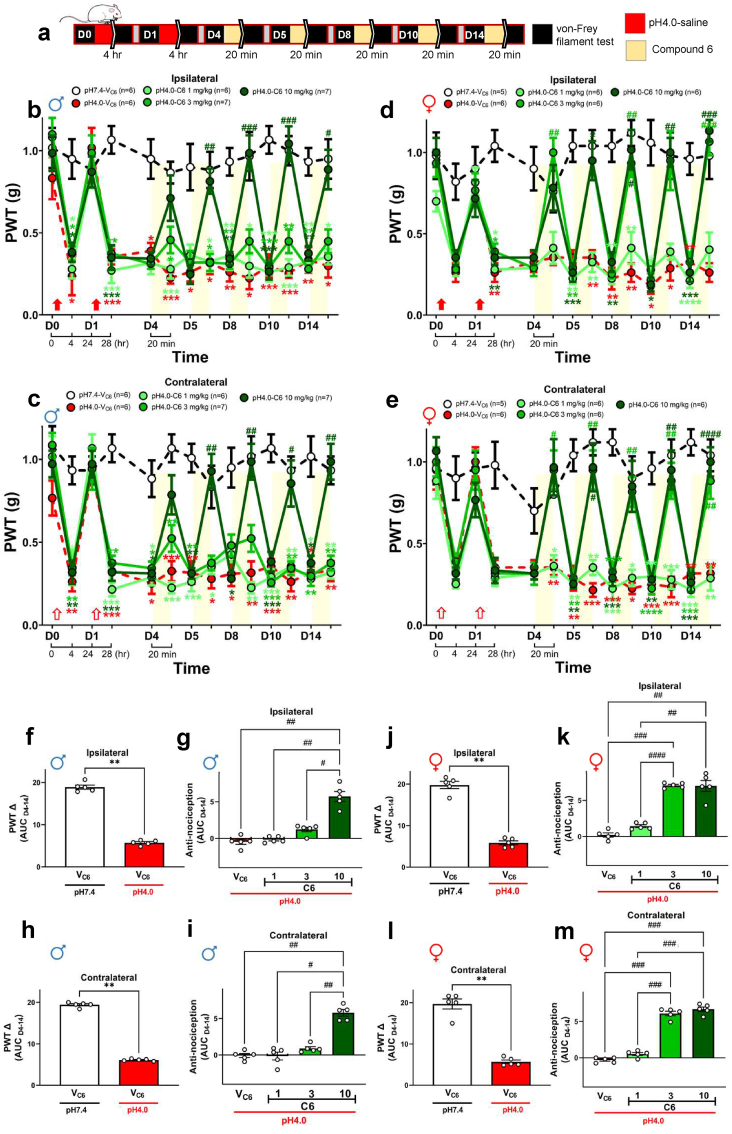
**Effects of systemic Compound 6 on mechanical allodynic response induced by dual acidic-saline injection (ASI) in male and female ICR mice.** a: Experimental timeline. ASI was bilaterally injected (*i.m.*) into ICR mice on day 0 and day 1, 24 h apart (red arrows). The paw withdrawal threshold (PWT) to von-Frey filament stimulation was assessed before and 4 h after the 1st and 2nd acidic-saline injections, as well as, on the denoted day, before and 20 min after *i.p*. administration of Compound 6 (1, 3, 10 mg/kg). b–e: The time course of PWT in ipsilateral (b, d) and contralateral (c, e) sides in male (b, c) and female (d, e) mice. f, h, j, l: Summated area under the curve (AUC) of PWT changes (PWT Δ) across day 4–14 in ASI and saline-control groups. g, i, k, m: Anti-nociception effects were quantified as the cumulative net PWT AUC across days 4–14, referenced to the daily baseline PWT in each treatment group. ∗P < 0.05, ∗∗P < 0.01, ∗∗∗P < 0.001, ∗∗∗∗P < 0.0001, vs. pH7.4-V_C6_; ^#^P < 0.05, ^##^P < 0.01, ^###^P < 0.001, ^####^P < 0.0001 vs. pH4.0-V_C6_. Two-way repeated-measures ANOVA with Bonferroni post-hoc test (b-e). ∗∗P < 0.01. Mann–Whitney test (f, h, j, l). ^#^P < 0.05, ^##^P < 0.01, ^###^P < 0.001, ^####^P < 0.0001. One-way ANOVA with Tukey post-hoc test (g, i, k, m). All data are mean ± SEM with the n number indicated in parentheses.

PWTs were measured in the tested mouse before (baseline PWT), and 4 h after the first acidic-saline injection on day 0. Also, PWTs were measured before the second acidic-saline injection on day 1 (24 h after the first acidic-saline injection), and 4 h afterward (Fig. 1a). Thereafter, mice were assessed for chronic mechanical allodynia, shown by decreased a PWT compared to baseline, 4–14 days after the first acidic-saline injection.

The maximal possible effect (MPE%) was calculated, to evaluate the dose-dependency of the anti-allodynic effect of the tested drug, with the formula: MPE% = (post-drug PWT – pre-drug PWT)/(cut-off von-Frey – pre-drug PWT) ∗ 100. The cut-off von-Frey filament was set at 2.0 g.

#### The ICS model

The ICS model was established as previously described [28], with modifications. At day 0, mice (two per cage) were placed on a rectangular welded stainless-steel mesh within a clear Plexiglas container (12.5 cm × 15 cm x 24 cm), containing food and agar. At 4:30 p.m., mice were transferred in their container to a 4 °C cold room and remained there overnight. At 10:00 a.m., on day 1, mice were transferred back to a 24 °C room. After 30 min, the mice returned back to the cold room for 30 min. This procedure was repeated every 30 min until 4:30 p.m. Mice then remained in 4 °C overnight. The same treatment was repeated daily until day 5 at 10:00 a.m. Control mice remained in the 24 °C room for the 5-day period. Mice were assessed for mechanical allodynia and thermal hyperalgesia by their PWT and PWL using von-Frey and Hargreaves assessments, respectively, at day 0 and days 10–20. The data from both hind paws was collected and analyzed.

### Anxiety-like behaviors

#### Elevated plus maze test

We measured anxiety-like behaviors of mice by elevated plus maze (EPM) [34] and light/dark box [35] tests. The EPM apparatus, consisting of two open arms (30 cm × 5 cm) and two closed arms (30 cm × 5 cm x 25 cm) extending from the center intersection zone (5 cm × 5 cm), was elevated 50 cm above the floor. Mice were placed in the center zone and then allowed to freely explore for 10 min. The mouse was tracked by a video camera placed above the maze, and the video was analyzed through the ToxTrac program [36]. Anxiety-like behavior was considered in mice which remained in the closed arm significantly longer than in the open arm, performed significantly fewer entries into the open arm, or traveled a significantly less distance in the open arm. Open arm time was measured by ([open arm time]/[total arm time] ∗ 100). Open arm distance was measured by (([open arm distance]/[total arm distance]) ∗ 100). Open arm entries were counted manually.

#### Light dark box test

The light-dark box apparatus consisted of light and dark two compartments (50 cm × 25 cm x 30 cm) connected to the center zone with a single opening to each compartment. Mice were individually placed at the center zone and left undisturbed for 10 min. Mice exploratory behaviors between the two compartments were recorded. Anxiety-like behavior was considered in mice which remained in the dark box significantly longer than in the light box, and had a reduced number of box transitions.

### Immunofluorescence

Mice were sacrificed by *trans*-cardiac perfusion using phosphate-buffered saline (pH7.4) followed by paraformaldehyde (4% PFA) whilst under anesthesia. The spinal cord was then harvested, and the L3-L5 segment was excised with respective DRG after overnight paraformaldehyde post-fixation at 4 °C. After dehydration in 30% sucrose-PBS for 2–5 days, tissues were embedded with the Optimal Cutting Temperature (OCT) compound. Spinal cord transverse sections (20 μm) or DRG coronal sections (12 μm) were then cut using a cryostat (Leica CM3050S, Leica Biosystems, Illinois, USA) to be used in immunofluorescent staining. The cerebellum was also collected, and sagittal sections (30 μm) were cut using a cryostat to be used as a positive control.

Triple immunofluorescent co-staining of the α6 subunit (the α6GABA_A_R marker) was performed with isolectin B4 (IB4) (a non-peptidergic sensory neuronal marker) and neuronal nuclei (NeuN) (a neuronal marker), or with cellular Fos (c-Fos) (a neuronal activity marker) and 4’,6-diamidino-2-phenylindole (DAPI) (a nuclear specific cell marker) in the L3-L5 spinal cord. When testing c-Fos, a positive signal was identified by a uniform hoop like structure. Mice were injected with vehicle or Compound 6 10 mg/kg, 20 min before von-Frey testing, and then sacrificed 90 min after repeated von-Frey fiber application. Triple immunofluorescent staining of the α6 subunit was also performed in DRG sections with IB4 and N-52 (a myelinated neuronal marker). Double immunostaining of the α6 subunit and DAPI was performed on cerebellum sections. Sections were washed three times with phosphate-buffered saline (PBS) (5 min each wash), and then blocked by goat serum (10%) and Triton-X (0.3%) for 2 h at room temperature. After blocking, slides were incubated with primary antibodies overnight at 4 °C. Primary antibodies utilized in this study were rabbit-*anti*-GABA_A_ receptor α6 (1:50, Novus, cat no. NB300–196), mouse-*anti*-NeuN (1:200, cat no. MAB377), anti-IB4-fluorescein isothiocyanate conjugated (4 ng/ml, cat no. I21411), mouse-*anti*-Neurofilament 200 kDa Antibody, clone N-52 (1:200, cat no. MAB5266), and mouse-*anti*-c-Fos (E−8) (1:400, cat no. sc-166940). Secondary antibody staining was performed at room temperature for 2 h using goat anti-rabbit 594, and goat anti-mouse 647. DAPI was also used to stain nuclear bodies.

All images were acquired using the Confocal microscope (ZEISS LSM 880) at 10x and 20x magnifications, and analyzed using ZEN software with the same laser intensity, gain, and threshold setting for each tissue. For each mouse, 3–4 random sections of each tissue were imaged. To measure the fluorescence intensity, Image J software was used. Fluorescence intensity was measured as mean of integrated density in the region of interest normalized by the background in Arbitrary units (AU)/μm2 using the formula: (mean ∗ area) - (background mean ∗ area) / area.

### Drugs

Two PQs, Compound 6 [2,5-dihydro-7-methoxy-2-(4-methoxyphenyl)-3H-pyrazolo[4,3-c]quinolin-3-one] and DK-I-56-1 [7-methoxy-2-(4-methoxy-d3-phenyl)−2,5-dihydro-3H-pyrazolo-[4,3-c]quinolin-3-one], were synthesized as reported in our previous study [22]. Furosemide and gabapentin were purchased from Tocris Bioscience (Bristol, UK).

Furosemide and PQs were each dissolved in a vehicle containing 20% dimethyl sulfoxide, 20% Kolliphor EL, and 60% saline. Gabapentin was dissolved in normal saline. For intraperitoneal (*i.p.*) injection, the injection volume was 10 ml/kg. For intrathecal (*i.t.*) furosemide administration, mice were anesthetized by 2% isoflurane, and 5 μL of the tested drug or its vehicle was injected into the subarachnoid space between the lumbar 4 and lumbar 5 vertebrae. A successful *i.t* injection was considered in mice which showed a tail flick [37]. All drug doses and treatment times used in this study were selected based on pilot studies or due to having previously been shown to have significant *in vivo* activity in mice [23,24,31,[37], [38], [39]].

### Data analysis and statistics

Nociceptive responses were assessed by the PWT for mechanical allodynia and the PWL for thermal hyperalgesia at each tested day and time point. The area under curve (AUC) of PWTs over the tested period was calculated using the trapezoidal method, setting the baseline at 0, and total AUC was plotted as the PWT change (PWT Δ). To evaluate the anti-nociceptive effect of the tested drug, the net AUC of PWT or PWL over the tested period was measured with the baseline set for each individual mouse, using the respective PWT or PWL obtained before drug treatment, on the tested day.

Data are presented as mean ± S.E.M. Multiple data sets were analyzed using two-way analyses of variance (ANOVA) or one-way ANOVA, with Bonferroni or Tukey post hoc testing to correct for multiple comparisons, unless otherwise stated in figure legends. Two-way ANOVA with repeated measures over time was used for the time-course data. Significance was set at ∗P, 0.05, ∗∗P, 0.01, ∗∗∗P, 0.001, and ∗∗∗∗P, 0.0001 levels.

## Results

### Effects of PQs in the ASI model

#### Compound 6 (*i.p.*) attenuated ASI-induced mechanical allodynia without tolerance, with greater sensitivity in females

First, to model long-lasting mechanical allodynia, we established the ASI fibromyalgia model in ICR mice (Fig. 1a). Two acidic (pH4.0)-saline injections induced long-lasting mechanical allodynia on both ipsilateral (Fig. 1b–d) and contralateral (Fig. 1c–e) sides in both males (Fig. 1b and c) and females (Fig. 1d and e), indicated by markedly reduced PWTs, as compared with the normal saline (pH7.4)-injected group. Mechanical allodynia was present on each tested day from days 4–14 (red vs. open symbols, Fig. 1b–e).

To evaluate the therapeutic effects of PQs for the relief of ASI-induced mechanical allodynia, mice were repeatedly injected with Compound 6 (1, 3, 10 mg/kg, *i.p.*) or vehicle (V_C6_) from day 4. In males, Compound 6 at 10 mg/kg (*i.p.*) (dark green symbols), but not 1 mg/kg (light green symbols), restored the decreased PWTs to a level as in saline-injected mice (open symbols) (Fig. 1b and c), producing a significant anti-allodynic effect. At 3 mg/kg (green symbols), it induced a partial, but insignificant, anti-allodynic effect. Notably, the anti-allodynic effect of Compound 6, as measured by the MPE, did not gradually wane over the course of each test from days 4–14 (Supplementary Fig. s2a, b), indicating no drug tolerance developed. The overall PWT change (PWTΔ) across days 4–14 showed a significant decrease in bilateral PWTs of ASI mice (Fig. 1f and h). The summated net AUC showed that Compound 6 induced a bilateral anti-allodynic effect in ASI-treated male mice in a dose-dependent manner (Fig. 1g and i).

In females, ASI also markedly induced ipsilateral (Fig. 1d and j) and contralateral (Fig. 1e and l) mechanical allodynic responses. Compound 6 significantly increased PWTs in female ASI mice (Fig. 1d, e, k, m), back to the level as in the normal saline-control groups (Fig. 1d and e), at both 3 and 10 mg/kg, respectively. Notably, the maximal effective dose, 3 mg/kg, in females is lower than that (10 mg/kg) in males, suggesting that females are more sensitive to Compound 6. During repeated administrations, female mice also did not develop tolerance to the anti-allodynic effect of Compound 6 (Supplementary Fig. s2c, d). When comparing the anti-nociceptive effects of respective doses in male and female ASI mice, the effects of Compound 6 at 1 mg/kg and 3 mg/kg were significantly greater in females versus males (Supplementary Fig. s3a, b).

In addition to mechanical allodynia, anxiety is commonly reported in fibromyalgia patients [40]. In both male (Supplementary Fig. s4a-c) and female (Supplementary Fig. s4d-f) ICR mice, ASI induced anxiety-like behaviors in the EPM test. This was more prominent in females, as shown by a reduced open-arm time, open-arm distance, and open-arm entries. Compound 6 (10 mg/kg, *i.p.*) tended to, but insignificantly, reduce ASI-induced anxiety-like behaviors in males, by increasing open-arm time, and in females by increasing open-arm distance, however these differences were not statistically significant.

#### The anti-allodynic effect of Compound 6 is mediated by α6GABA_A_Rs

##### The effect of Compound 6 was abolished in *Gabra6*-KO mice

**Fig. 2:**
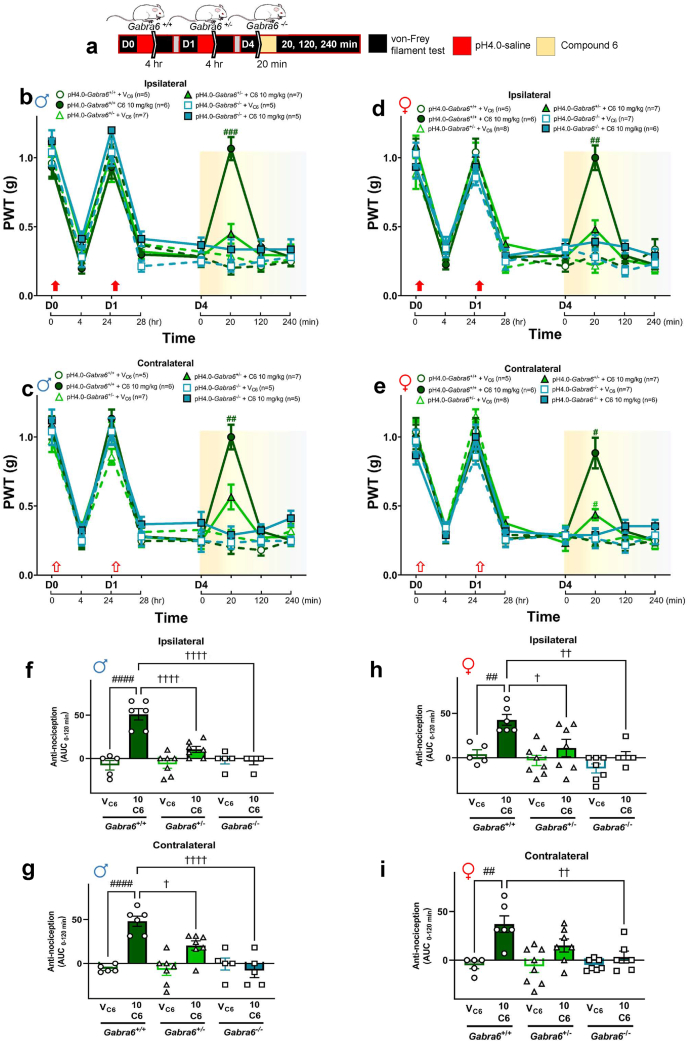
**Effect of systemic Compound 6 on ASI-induced allodynic responses in male and female *Gabra6*-knockout (KO) ICR mice.** a: Experimental timeline. ASI was conducted in three genotypes, *Gabra6*^+/+^, *Gabra6*^+/−^ and *Gabra6*^−/−^ mice with the same protocol as in Fig. 1. PWT was measured on day 4, before and 20–240 min after *i.p*. administration of Compound 6 (10 mg/kg). b–e: Time courses of bilateral PWT as described in Fig. 1. f–i: Anti-nociception effects were quantified as in Fig. 1, except the net AUC of PWT 0–120 min after Compound 6 administration was calculated. ^#^*P* < 0.05, ^##^*P* < 0.01, ^###^*P* < 0.001, *vs. Gabra6*^*+/+*^ pH4.0-V_C6_; ^#^*P* < 0.05, *vs. Gabra6*^*+/−*^ pH4.0-V_C6_. Two-way repeated-measures ANOVA with Bonferroni post-hoc test (b–e). ^##^*P* < 0.01, ^####^*P* < 0.0001; ^†^*P* < 0.05, ^††^*P* < 0.01, ^††††^*P* < 0.0001. One-way ANOVA with Tukey post-hoc test (f–i). All data are mean ± SEM with the n number indicated in parentheses.

To confirm the anti-allodynic effect of Compound 6 was mediated by α6GABA_A_Rs we next tested Compound 6 in *Gabra6*-KO mice which received ASI. The ASI-treated *Gabra6* ^*+/+*^, *Gabra6* ^*+/−*^, and *Gabra6* ^*−/−*^ mice were injected with Compound 6 (10 mg/kg, *i.p.*) or vehicle (V_C6_) at day 4, and PWT was measured from 0 to 240 min (Fig. 2a). In *Gabra6* ^*+/+*^ mice (dark green filled symbols), Compound 6 increased PWTs 20 min after administration, producing ipsilateral and contralateral anti-allodynic effects, which declined within 120 min in male (Fig. 2b and c) and female (Fig. 2d and e) mice. In contrast, in *Gabra6* ^−/−^ mice (blue symbols), Compound 6 did not affect PWTs, and failed to induce a significant anti-allodynic effect in both sexes. Whereas in *Gabra6* ^*+/−*^ mice (green symbols), Compound 6 elicited a slight anti-allodynic effect at 20 min, but the change was statistically insignificant (Fig. 2b–e), except at the contralateral side of female mice (Fig. 2e). Vehicle-treated mice showed significant bilateral mechanical allodynia in both sexes across all genotypes (open symbols, Fig. 2b–e). The basal ipsilateral or contralateral PWTs at day 0 did not differ significantly among three genotypes (Fig. 2b–e), suggesting endogenous GABA-α6GABA_A_R transmission does not significantly impact the physiological nociceptive response. Further PWT AUC analyses revealed bilateral anti-allodynic effects of Compound 6 occured significantly in *Gabra6* ^*+/+*^, but not *Gabra6* ^*−/−*^, mice, and slightly but insignificantly in *Gabra6* ^+*/−*^ mice (Fig. 2f–i), suggesting a slight gene dose-dependent trend.

#### Spinal α6GABA_A_Rs are key for the anti-allodynic effect of Compound 6

##### The effect of Compound 6 was antagonized by *i.t.* but not *i.p.* injection of furosemide, an α6GABA_A_R antagonist

**Fig. 3:**
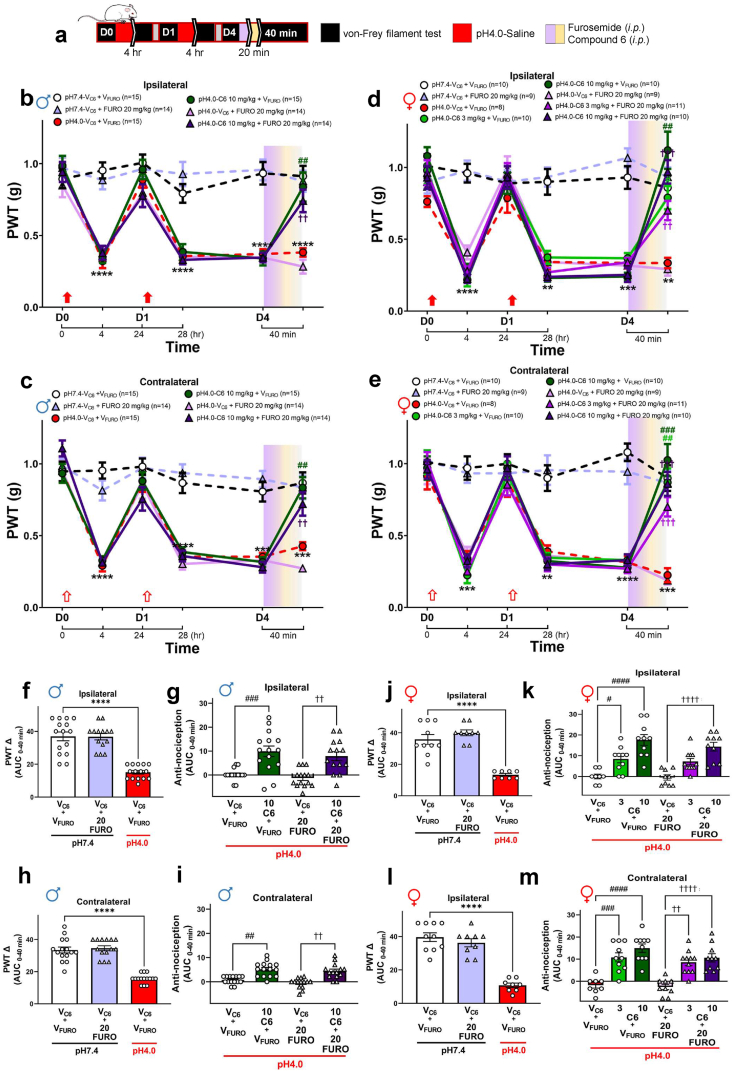
**Effect of peripheral α6GABA_A_ receptor blockade by *i.p*. furosemide on the anti-allodynic effects of Compound 6 in the ASI model.** a: Experimental timeline. ASI was performed in mice with the same protocol as in Fig. 1. On day 4, mice received furosemide (20 mg/kg) *i.p*. pretreatment for 20 min, followed by *i.p*. administration of Compound 6 (3 or 10 mg/kg). PWT was measured before drug treatment, and 20 min after Compound 6 administration, i.e., 40 min after furosemide administration. b–e: Time courses of bilateral PWT as described in Fig. 1. f–i: Anti-nociception effects were quantified as in Fig. 1, except the net AUC of PWT before furosemide and 20 min after Compound 6 administration was calculated. ∗∗*P* < 0.01, ∗∗∗*P* < 0.001, ∗∗∗∗*P* < 0.0001, *vs.* pH7.4-V_C6/FURO_; ^##^*P* < 0.01, ^###^*P* < 0.001, *vs.* pH4.0-V_C6/FURO_. Two-way repeated-measures ANOVA and Bonferroni post-hoc test (b–e). ∗∗∗∗*P* < 0.0001, ^#^*P* < 0.05, ^##^*P* < 0.01, ^###^*P* < 0.001, ^####^*P* < 0.0001, ^††^*P* < 0.01, ^††††^*P* < 0.0001. One-Way ANOVA with Tukey post-hoc test (f–m). All data are mean ± SEM with the n number indicated in parentheses.

To identify the location of α6GABA_A_Rs that mediated the anti-allodynic effect of Compound 6, we employed a pharmacological approach using furosemide. Furosemide is a BBB-impermeable [41] α6GABA_A_R-selective antagonist [29], and if administered systemically, would block peripheral, but not central, α6GABA_A_ receptors. Thus, we first investigated the PQ induced anti-allodynic effect of peripheral α6GABA_A_ receptors by *i.p.* administration of furosemide. In male ASI mice, *i.p.* pretreatment with furosemide (20 mg/kg) for 20 min on day 4 (Fig. 3a) did not significantly alter the PWT-restoring effect of Compound 6 (10 mg/kg, *i.p*.) (dark purple vs. green symbols, Fig. 3b, c, g, i). In the group without receiving Compound 6, *i.p.* furosemide also did not significantly affect the PWTs (light purple vs. red symbols, Fig. 3f and h).

In females, we pretreated ASI mice with furosemide against two doses of Compound 6 (3 and 10 mg/kg, *i.p*.), due to both doses previously showing good efficacy (Fig. 1d and e). As in males, furosemide did not affect the PWTs in the vehicle group, and slightly, but not significantly, inhibited the anti-allodynic effect of Compound 6 at 3 mg/kg (purple vs. green symbols) and 10 mg/kg (dark purple vs. dark green symbols) (Fig. 3d, e, k, m). Similarly, in non-ASI females, *i.p.* furosemide did not significantly affect PWTs (Fig. 3j and l). These results suggest that the anti-allodynic effect of Compound 6 is unlikely to be mediated by α6GABA_A_Rs expressed at the periphery, in both male and female mice.

##### Intrathecal furosemide pretreatment attenuated the anti-allodynic effect of Compound 6 in the ASI model

**Fig. 4:**
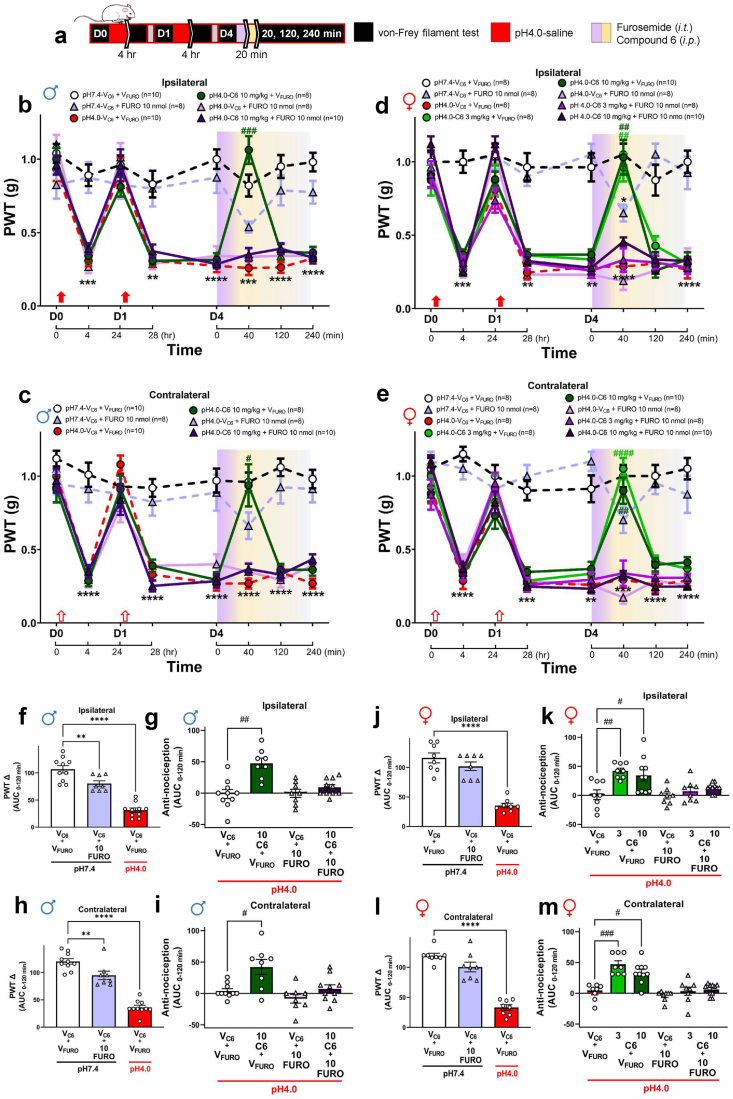
**Effect of central α6GABAA receptor blockade by i.t. furosemide on the anti-allodynic effects of Compound 6 in the ASI model.** a: Experimental timeline. ASI induction, PWT assessment and drug administration were the same as employed in Fig. 3, except that post-treatment PWT was measured for a longer time; 40, 120 and 240 min after furosemide administration on day 4. b–e: Time courses of bilateral PWT as described in Fig. 2. f–i: Summated PWT Δ AUC (f, h, j, l) and anti-nociception effects (g, i, k, m) across 0–120 min after drug administration were quantified. ∗*P* < 0.05, ∗∗*P* < 0.01, ∗∗∗*P* < 0.001, ∗∗∗∗*P* < 0.0001, *vs.* pH7.4-V_C6_ + V_FURO_; ^#^p < 0.05, ^##^*P* < 0.01, ^###^*P* < 0.001, ^####^*P* < 0.0001, *vs.* pH4.0-V_C6_ + V_FURO_. Two-way repeated-measures ANOVA and Bonferroni post-hoc test (b–e). ∗∗*P* < 0.01, ∗∗∗∗*P* < 0.0001; ^#^*P* < 0.05 ^##^*P* < 0.01, ^###^*P* < 0.001. One-way ANOVA with Tukey post-hoc test (f–m). All data are mean ± SEM with the n number indicated in parentheses.

To further explore whether the α6GABA_A_Rs identified in the spinal dorsal horn [42], a crucial pain-regulating region, are the anti-allodynic action site of Compound 6, we *i.t.* pretreated mice with furosemide (10 nmol) or vehicle (V_FURO_) 20 min before administration of Compound 6 or vehicle (V_C6_, *i.p.*) on day 4 (Fig. 4a). In male ASI mice which received *i.t.* V_FURO_, Compound 6 induced a significant bilateral anti-allodynic effect 20 min after administration, which lasted less than 2 h (red vs. dark green symbols) (Fig. 4b and c). However, in the *i.t.* furosemide-pretreated group, the anti-allodynic effect of Compound 6 (10 mg/kg, *i.p.*) was abolished (dark purple vs. dark green symbols). Furosemide (*i.t.*) did not significantly alter the PWTs in the group without receiving Compound 6 (light purple vs. red symbols). PWT AUC analyses at 0–120 min also supported that *i.t.* furosemide, at the dose without producing significant changes in PWTs of ASI mice, significantly prevented the anti-allodynic effect of Compound 6 (Fig. 4g and i). Notably, in control mice without receiving ASI, *i.t.* furosemide *per se* significantly reduced basal nociceptive responses (light blue vs. white bars, Fig. 4f and h), suggesting α6GABA_A_R-mediated GABAergic transmission in the spinal cord may perform an endogenous anti-nociceptive role.

In female ASI mice (Fig. 4d and e), *i.t.* furosemide prevented the anti-allodynic effect of Compound 6 at both 3 mg/kg (purple vs. green symbols) and 10 mg/kg (dark green vs. dark purple symbols). The PWT AUC analyses yielded statistically significant results consistent with these findings (Fig. 4k and m). Interestingly, with *i.t.* furosemide pretreatment, the PWT in Compound 6-treated mice with 10 mg/kg tended to be greater than those with 3 mg/kg (purple vs. dark purple bars, Fig. 4k and m), suggesting an antagonist interaction by furosemide at spinal α6GABA_A_Rs, which are critical for the anti-allodynic effect of Compound 6. Similarly, as in males, *i.t.* furosemide reduced basal nociceptive responses in females, with significance at the ipsilateral side (light blue vs. white bars, Fig. 4d, e, j, l).

#### The α6 subunit immunofluorescent staining in the spinal dorsal horn and DRG

##### The α6 subunit expression in the spinal dorsal horn

**Fig. 5:**
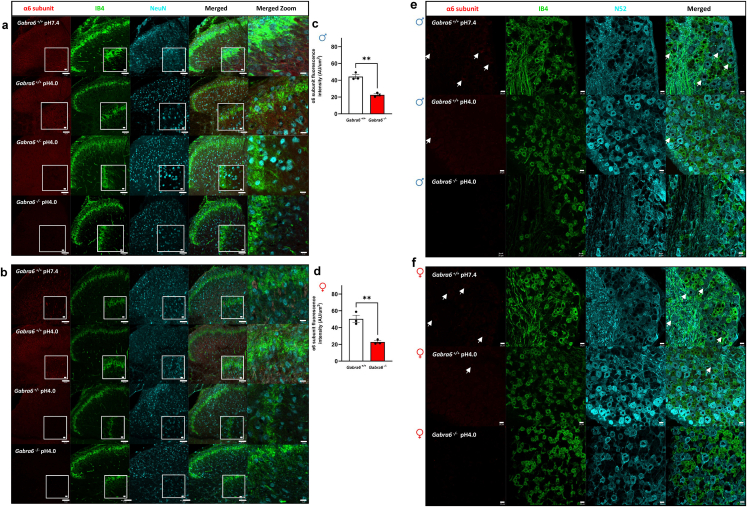
**Expression of the α6 subunit representing the α6GABA_A_R at the spinal cord and DRG of *Gabra6*-wild-type and *Gabra6*-knockout ICR male and female mice with ASI.** a–f: Immunofluorescence staining of the α6 subunit in the spinal cord dorsal horn (a, b, c, d) or DRG (e, f), and at the ipsilateral side of non-ASI or ASI male (a, c, e) and female (b, d, f) *Gabra6*^+/+^, *Gabra6*^+/−^ and *Gabra6*^−/−^ mice. Spinal cord dorsal horn sections were stained with the α6 subunit (red), IB4 (green) and NeuN (blue). Statistical analysis of the α6 subunit fluorescence intensity was performed in both male and female *Gabra6*^+/+^ and *Gabra6*^−/−^ mice using the student t-test (c, d). DRG sections were stained with the α6 subunit (red), IB4 (green) and N-52 (blue). The n = 3 mice per group. Scale bars represent 50 μm and 20 μm for spinal cord and DRG sections, respectively.

Given that *i.t.* furosemide robustly antagonized the anti-allodynic effect of Compound 6, whilst *i.p.* furosemide marginally affected it, α6GABA_A_Rs in the spinal cord are likely the main action target of Compound 6. We therefore examined the expression of α6GABA_A_Rs in the spinal cord dorsal horn and DRG by immunofluorescent staining of the α6 subunit. The specificity of the α6 subunit antibody was validated using *Gabra6* ^−/−^ mice, with cerebellar tissue serving as a positive control. As shown in Supplementary Figure s5, the α6 subunit fluorescence (red) in the cerebellar granule layer in the *Gabra6* ^*+/+*^ group was absent in the *Gabra6* ^*−/−*^ group (Supplementary Fig. s5a), evidenced by a markedly reduced fluorescence intensity in the knockout group (Supplementary Fig. s5b). In spinal cord sections, the α6 subunit was co-localized with NeuN, a neuronal marker, in male (Fig. 5a) and female groups (Fig. 5b). Similarly, in *Gabra6* ^*+/+*^ mice, the α6 subunit fluorescence intensity was significantly greater than in *Gabra6* ^*−/−*^ mice, for both males and females, respectively (Fig. 5c and d). In addition, we found that the c-Fos signal, a neuronal activation marker, increased in the spinal dorsal horn neurons of vehicle-treated ASI male and female mice, whilst Compound 6 reduced c-Fos expression (Supplementary Fig. s6a, b). These results suggest that α6GABA_A_Rs are present in spinal dorsal horn neurons and that Compound 6 reduces the increased activity of these neurons under mechanical stimulation in ASI mice.

Notably, the α6 subunit also partially co-localized with a few spots and fibers with positive immunoreactivity of IB4, a marker of non-peptidergic primary sensory neurons [43], suggesting the presence of α6GABA_A_Rs on the central terminals of non-peptidergic primary sensory neurons. We were unable to detect a significant and strong α6 subunit signal in DRG sections of male (Fig. 5e) and female mice (Fig. 5f). However, in a small percentage of neurons, typically with large diameter and stained by N-52, a marker of myelinated sensory neurons [44], the α6 subunit showed a weak signal.

#### Benchmarking PQs against gabapentinoids in ASI mice

##### The efficacy and duration of DK-I-56-1 was comparable to gabapentin

**Fig. 6:**
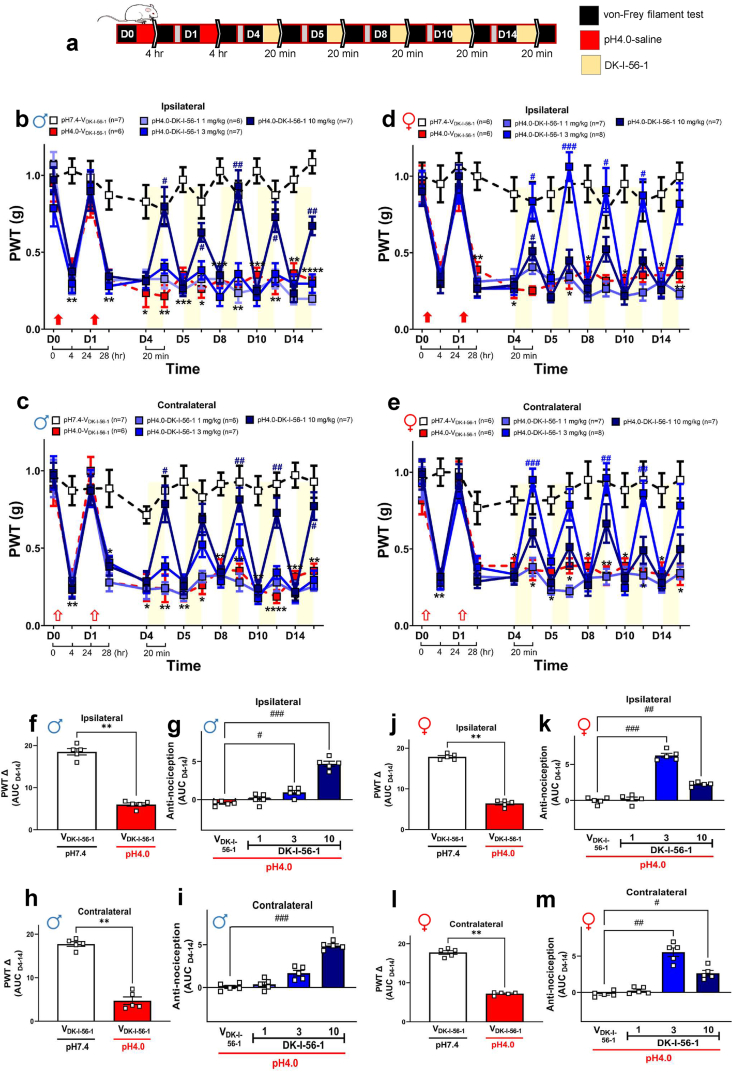
**Effects of systemic DK-I-56**–**1 on mechanical allodynic responses induced by ASI.** a: Experimental timeline. ASI induction, PWT assessment and drug administration were the same as employed in Fig. 1, except DK-I-56-1 (1, 3, 10 mg/kg, *i.p.*) was administered. b–e: Time courses of bilateral PWT as described in Fig. 1. f–i: Anti-nociception effects were quantified as in Fig. 1. ∗*P* < 0.05, ∗∗*P* < 0.01, ∗∗∗*P* < 0.001 ∗∗∗∗*P* < 0.0001, *vs.* pH7.4-V_DK-I-56-1_; ^#^*P* < 0.05, ^##^*P* < 0.001, ^###^*P* < 0.001, *vs.* pH4.0-V_DK-I-56-1_. Two-way repeated-measures ANOVA and Bonferroni post-hoc test (b–e). ∗∗*P* < 0.01. Mann–Whitney test (f, h, j, l). ^#^*P* < 0.05, ^##^*P* < 0.01, ^###^*P* < 0.001. One-way ANOVA with Tukey post-hoc test (g, i, k, m). All data are mean ± SEM with the n number indicated in parentheses.

We also examined effects of DK-I-56-1, a deuterated analogue of Compound 6, with a longer half-life and higher oral bioavailability [22], using ASI mice (Fig. 6a). Systemic DK-I-56-1 treatment restored the reduced ipsilateral (Fig. 6b and d) and contralateral (Fig. 6c and e) PWTs in male (Fig. 6b, c, f, h, g, i) and female (Fig. 6d, e, j, l, k, m) ASI mice. Similar to the results with Compound 6, female mice were more sensitive to DK-I-56-1, with mechanical allodynia abolished at 3 mg/kg and 10 mg/kg, respectively, without the development of drug tolerance after repeated administrations (Supplementary Fig. s7a-d). When comparing the anti-nociception effect of respective doses in male and female ASI mice, the effects of DK-I-56-1 at 3 mg/kg were significantly greater in females versus males (Supplementary Fig. s8a, b).

**Fig. 7:**
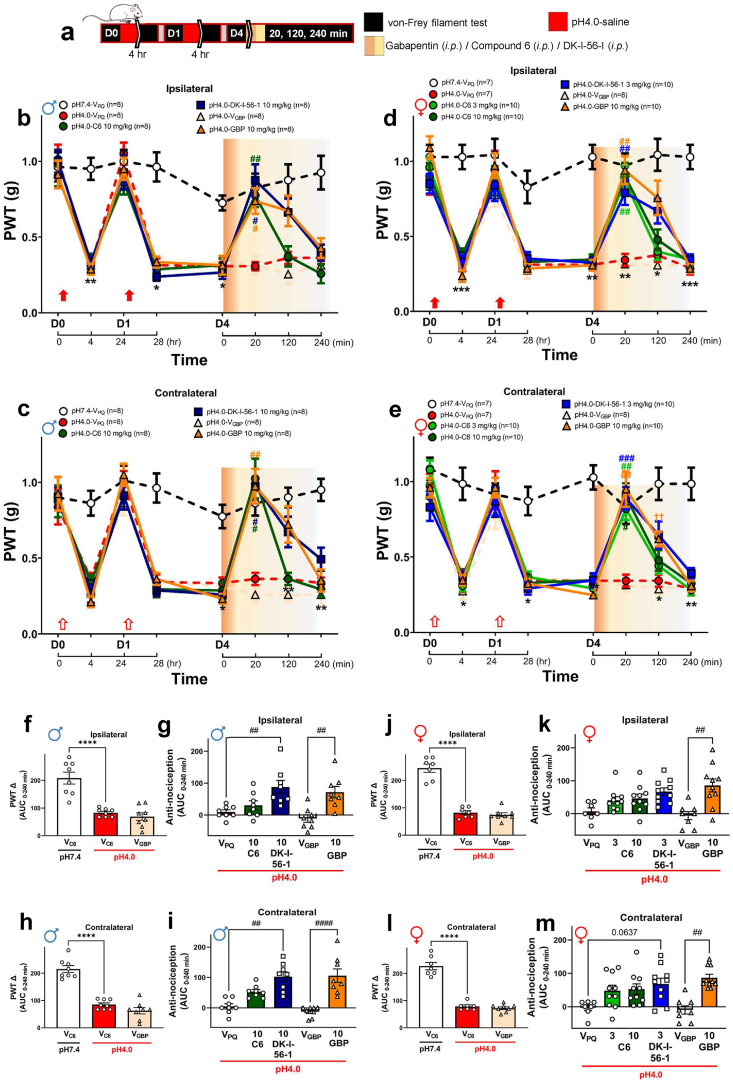
**Benchmarking effects and durations of PQs against gabapentin for mechanical allodynic responses induced by ASI.** a: Experimental timeline. ASI was performed in mice with the same protocol as in Fig. 1. On day 4, PWT was measured before, and 20–240 min after *i.p*. administration of Compound 6 (3 or 10 mg/kg), DK-I-56-1 (3 or 10 mg/kg) or gabapentin (10 mg/kg). b–e: Time courses of bilateral PWT as described in Fig. 2, except from 0 to 240 min ∗*P* < 0.05, ∗∗*P* < 0.01, ∗∗∗*P* < 0.001, *vs.* pH7.4-V_PQ_; ^#^*P* < 0.05, ^##^*P* < 0.01, ^###^*P* < 0.001, *vs.* pH4.0-V_PQ_; ^#^*P* < 0.05, ^##^*P* < 0.01, *vs.* pH4.0-V_GBP_. Two-way repeated-measures ANOVA and Bonferroni post-hoc test. f–m: Summated PWT Δ AUC (f, h, j, l) and anti-nociception effects (g, i, k, m) were quantified as in Fig. 4. ∗∗∗∗*P* < 0.0001; ^##^*P* < 0.01, ^####^*P* < 0.0001. One-way ANOVA with Tukey post-hoc test (f–k). All data are mean ± SEM with the n number indicated in parentheses.

Next, we benchmarked PQs against gabapentinoids, clinically used pain-reliving agents for fibromyalgia [30]. The anti-allodynic effects of PQs on day 4 at minimal effective doses, 10 mg/kg and 3 mg/kg, in males and females, were compared with that of gabapentin (10 mg/kg, *i.p.*) using both sexes (Fig. 7a). In males, Compound 6, DK-I-56-1 and gabapentin at 10 mg/kg produced significantly anti-allodynic effects at both ipsilateral and contralateral sides (Fig. 7b and c) without statistically significant differences when assessed 20 min after administration (*P* > 0.9999). Nevertheless, their action durations were different. The effect of Compound declined within 120 min, as observed in Fig. 4, whereas the effects of DK-I-56-1 and gabapentin lasted until 240 min (Fig. 7b and c). The summated PWT AUC at 0–240 min showed that the mechanical allodynia induced by ASI (Fig. 7f and h) was significantly rescued by DK-I-56-1 and gabapentin at ipsilateral and contralateral sides (Fig. 7g and i), without significant difference between treatment groups.

In female ASI mice, Compound 6 at 3 and 10 mg/kg, DK-I-56-1 at 3 mg/kg, and gabapentin at 10 mg/kg, significantly attenuated mechanical allodynia (Fig. 7d and e). No significant difference between Compound 6, DK-I-56-1, and gabapentin was observed at 20 min (*P* > 0.9999). At 120 min, the anti-allodynic effect of Compound 6 subsided. In contrast, DK-I-56-1 and gabapentin continued to produce an anti-allodynic effect lasting for at least 240 min. The summated AUC for PWTs at 0–240 min, showed ASI induced bilateral mechanical allodynia (Fig. 7j and l). Gabapentin significantly rescued mechanical allodynia (orange vs. open bars, Fig. 7k and m). Although the rescue effects of Compound 6 and DK-I-56-1 were not significant, there was no significant difference among three drug-treatment groups.

##### DK-I-56-1 and gabapentin produced an additive anti-allodynic effect at low doses

**Fig. 8:**
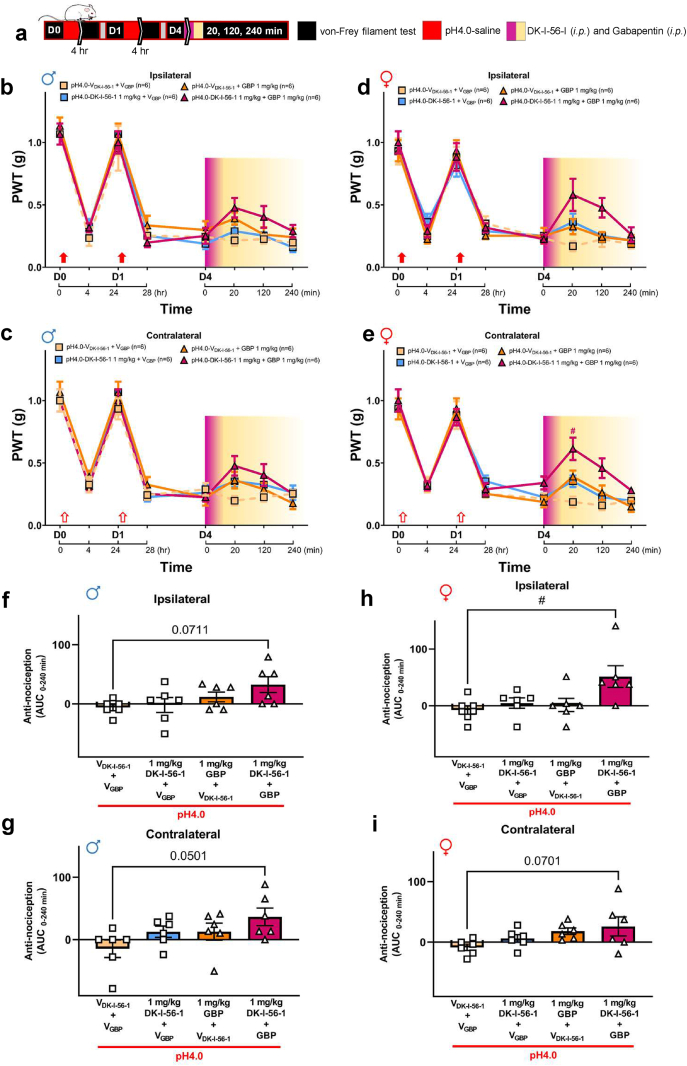
**Evaluating the anti-allodynic effect of combined DK-I-56**–**1 and gabapentin polytherapy against monotherapy.** a: Experimental timeline. ASI induction, PWT assessment and drug administration were the same as employed in Fig. 6, except DK-I-56-1 (1 mg/kg) and gabapentin (1 mg/kg) alone or in combination were administered. b–e: Time courses of bilateral PWT. ^#^*P* < 0.05, *vs.* pH4.0-V_DK-I-56-1_ + V_GBP_. Two-way repeated-measures ANOVA and Bonferroni post-hoc test (b–e). f–i: Anti-nociception effects in various groups. ^#^*P* < 0.05_._ One-way ANOVA with Tukey post-hoc test (f–i). All data are mean ± SEM with the n number indicated in parentheses.

Low dose polytherapy is commonly used in fibromyalgia patients to manage symptoms [45] by reducing the side effect risk and increasing therapeutic efficacy. We combined DK-I-56-1 (1 mg/kg) and gabapentin (1 mg/kg) at low doses to explore a possible additive anti-allodynic effect in ASI mice (Fig. 8a). In males, DK-I-56-1 or gabapentin alone induced a slight but insignificant anti-allodynic effect (Fig. 8b, c, f, g). However, combined treatment of DK-I-56-1 and gabapentin showed a trend to have a greater anti-allodynic effect than individual treatment groups.

In female mice, the DK-I-56-1 (1 mg/kg) and gabapentin (1 mg/kg) combination produced a significant anti-allodynic effect in the contralateral side at 20 min (Fig. 8e). A slight, although insignificant, anti-allodynic effect was also observed at the ipsilateral side (Fig. 8d). When calculating the AUC at 0–240 min, a significant anti-allodynic effect was observed at the ipsilateral side (Fig. 8h), and a slight but not significant effect at the contralateral side (Fig. 8i). Thus, in both male and female mice, polytherapy with DK-I-56-1 and gabapentin exceeded the anti-allodynic effect induced by DK-56-1 and gabapentin monotherapy.

### Therapeutic effects of PQs in ICS-treated ICR mice

#### Compound 6 attenuated mechanical allodynia in a mouse model of ICS

**Fig. 9:**
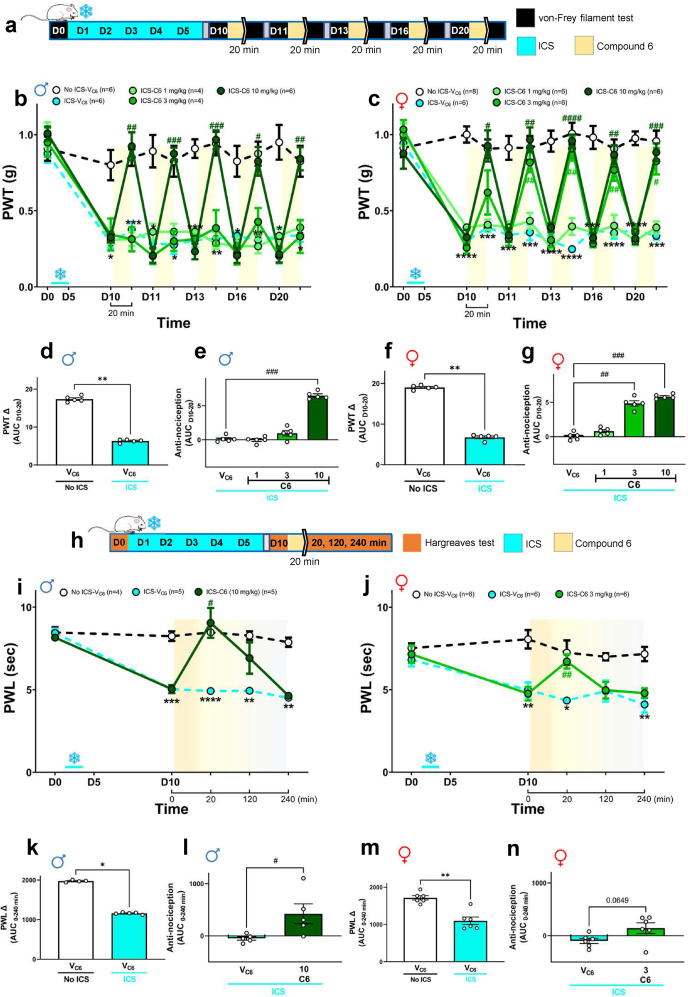
**Effects of Compound 6 on mechanical allodynia and thermal hyperalgesia in a fibromyalgia model induced by intermittent cold stress (ICS) in male and female ICR mice.** a: Experimental timeline. Mice were placed at 4 °C on days 0–5 and were transferred to 24 °C for 30 min intermittently for 6.5 h daily, and returned to 24 °C on day 5 (blue bars). The PWT to von-Frey filament stimulation was assessed before and on day 10 after ICS, as well as, on the denoted day, before and 20 min after *i.p*. administration of Compound 6 (1, 3, 10 mg/kg). b, c: Time courses of bilateral PWT as described in Fig. 1, except the average PWT taken from both hind paws was plotted. ∗*P* < 0.05, ∗∗*P* < 0.01, ∗∗∗*P* < 0.001, ∗∗∗∗*P* < 0.0001, *vs.* No ICS-V_C6_; ^#^*P* < 0.05, ^##^*P* < 0.01, ^###^*P* < 0.001, ^####^*P* < 0.0001, *vs.* ICS-V_C6_. Two-way repeated-measures ANOVA and Bonferroni post-hoc test. d–g: Summated AUC of PWT Δ (d, f) and anti-nociception effects (e, g), were quantified from days 10–20 as in Fig. 1. ∗∗*P* < 0.01. Mann–Whitney test (d, f). ^##^*P* < 0.01, ^###^*P* < 0.001. One-way ANOVA with Tukey post-hoc test (e, g). h: Experimental timeline. The paw withdrawal latency (PWL) to the Hargreaves device thermal stimulation was assessed before and on day 10 after ICS, and at 20–240 min after *i.p*. administration of Compound 6 (3 or 10 mg/kg). i, j: Time courses of bilateral PWL were calculated as described for PWT in Fig. 1. k–n: Summated AUC of PWT Δ (k, m) and anti-nociception effects (l, n), were quantified from days 10–20 as in Fig. 1. ∗*P* < 0.05, ∗∗*P* < 0.01, ∗∗∗*P* < 0.001, ∗∗∗∗*P* < 0.0001, *vs.* No ICS-V_C6_; ^#^*P* < 0.05, ^##^*P* < 0.01, *vs.* ICS-V_C6_. Two-way repeated-measures ANOVA and Bonferroni post-hoc test (i, j). ∗*P* < 0.05, ∗∗*P* < 0.01; ^#^*P* < 0.05. Mann–Whitney test (k–n). All data are mean ± SEM with the n number indicated in parentheses.

To further validate the effectiveness of α6GABA_A_R PAMs across different fibromyalgia animal models, we examined effects of PQs in mice exposed to ICS (Fig. 9a). In male mice, Compound 6 at 10, but not 1 and 3, mg/kg, significantly attenuated ICS-induced mechanical allodynia (Fig. 9b, d, e). In female ICS-mice, Compound 6, as in ASI-mice, also displayed a significant anti-allodynic effect at 3 and 10, but not 1, mg/kg (Fig. 9c, f, g). The anti-allodynic effect of Compound 6 did not develop tolerance in either sex from days 10–20 (Supplementary Fig. s9a, b). The anti-nociceptive effect of Compound 6 at 3 mg/kg in ICS mice was significantly higher in females than males (Supplementary Fig. s10a).

In addition to mechanical allodynia, ICS can also induce thermal hyperalgesia, lasting for an estimated 15 days [28]. Thus, we also assessed the effect of Compound 6 in thermal hyperalgesia on day 10 (Fig. 9h). Consistent with the results in response to mechanical allodynia, ICS-induced thermal hyperalgesia (Fig. 9k and m) was abolished 20 min after administration of Compound 6, at 10 mg/kg in males (Fig. 9i and l) and 3 mg/kg in females (Fig. 9j and n).

We also examined ICS-induced anxiety-like behaviors, which were previously reported in Swiss mice [46], by EPM (Supplementary Fig. s11a-f) and light and dark box (Supplementary Fig. s11g-j) tests on day 10. However, we failed to observe significant anxiety-like behaviors in both tests (Supplementary Fig. s11), except in female mice, which showed a significant decrease in open-arm distance traveled (Supplementary Fig. s11e).

#### Intrathecal furosemide pretreatment significantly attenuated the anti-allodynic effect of Compound 6 in ICS mice

**Fig. 10:**
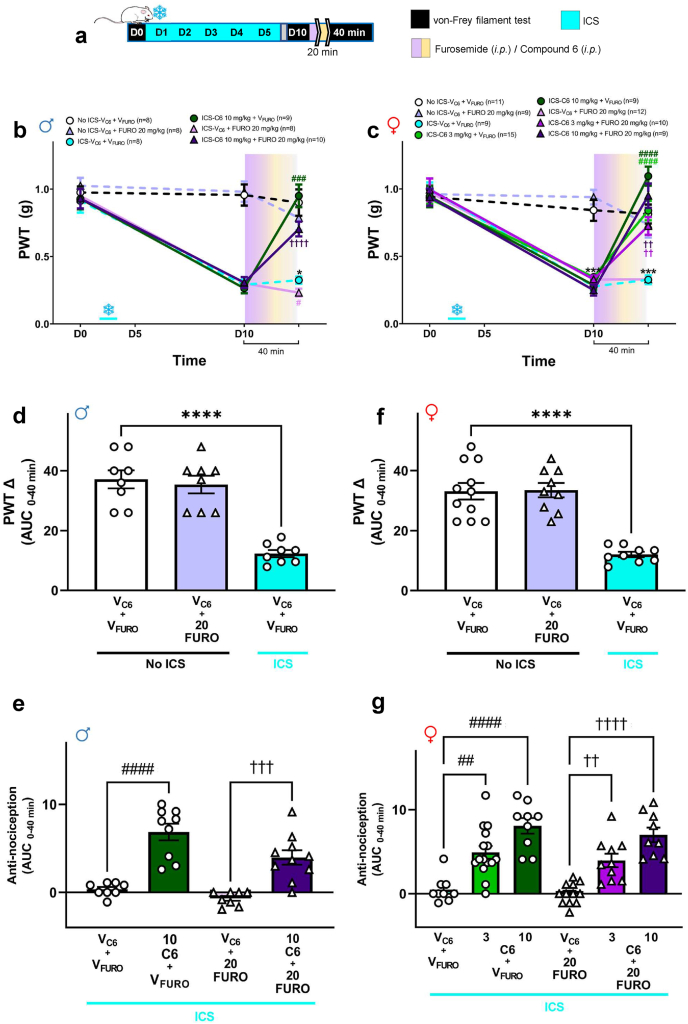
**Effect of peripheral α6GABA_A_ receptor blockade by *i.p.* furosemide on the anti-allodynic effects of Compound 6 in the ICS model.** a: Experimental timeline. ICS was performed in mice with the same protocol as in Fig. 8. On day 10, furosemide (20 mg/kg) was *i.p*. pretreated for 20 min, followed by *i.p*. administration of Compound 6 (3 or 10 mg/kg). PWT was measured before drug treatment, and 20 min after Compound 6 administration, i.e., 40 min after furosemide administration. b, c: Time courses of bilateral PWT as described in Fig. 8. d–g: Anti-nociception effects were quantified as in Fig. 8, except AUC of PWT before furosemide and 20 min after Compound 6 administration was measured. ∗*P* < 0.05, ∗∗∗*P* < 0.001, *vs.* No ICS-V_C6/FURO_; ^#^*P* < 0.05, *vs.* No ICS-V_C6_ + FURO 20 mg/kg; ^###^*P* < 0.001, ^####^*P* < 0.0001, *vs.* ICS-V_C6/FURO_; ^††^*P* < 0.01, ^††††^*P* < 0.0001, *vs.* ICS-V_C6_ + FURO 20 mg/kg. Two-way repeated-measures ANOVA and Bonferroni post-hoc test (b, c). ∗∗∗∗*P* < 0.0001; ^##^*P* < 0.01, ^####^*P* < 0.0001; ^††^*P* < 0.01, ^†††^*P* < 0.001, ^††††^*P* < 0.0001. One-way ANOVA with Tukey post-hoc test (d–g). All data are mean ± SEM with the n number indicated in parentheses.

Similar to the pharmacological approach in the ASI model, we pretreated ICS-challenged mice on day 10 with *i.p.* or *i.t.* injection of furosemide, respectively, before Compound 6 administration to distinguish the contribution of peripheral and central α6GABA_A_Rs in its anti-allodynic effect in this model. In the group *i.p.* pretreated with furosemide (20 mg/kg) (Fig. 10a), Compound 6 showed a significant anti-allodynic effect, measured at 40 min, not significantly different from that in the group pretreated with Compound 6 and *i.p.* V_FURO_, in either male (Fig. 10b, d, e) or female (Fig. 10c, f, g) mice. Furosemide (*i.p.*) did not affect PWTs in non-ICS mice (Fig. 10d and f). These results indicate that peripherally expressed α6GABA_A_Rs may not significantly contribute to the anti-allodynic effect of Compound 6 in ICS mice.

**Fig. 11:**
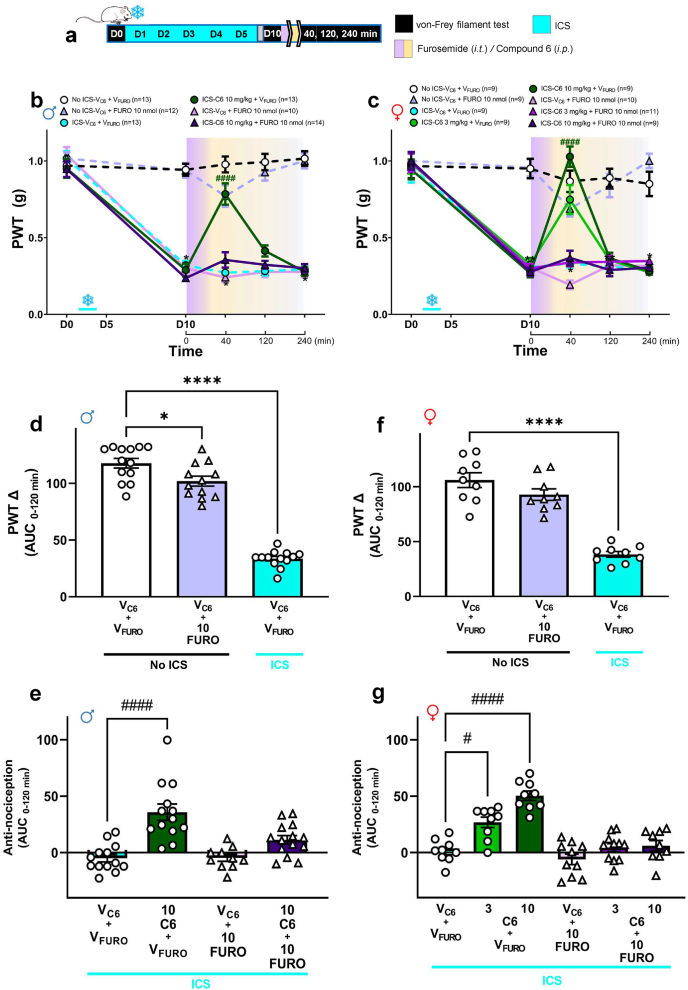
**Effect of central α6GABA_A_ receptor blockade by *i.t.* furosemide on the anti-allodynic effects of Compound 6 in the ICS model.** a: Experimental timeline. ICS induction, PWT assessment and drug administration were the same as employed in Fig. 9, except furosemide (10 nmol) was *i.t.* administered. b, c: Time courses of bilateral PWT. d–g: Anti-nociception effects were quantified as in Fig. 9. ∗*P* < 0.05, ∗∗*P* < 0.01, *vs.* No ICS-V_C6_ + V_FURO_; ^####^*P* < 0.0001, *vs.* ICS-V_C6_ + V_FURO_. Two-way repeated-measures ANOVA and Bonferroni post-hoc test (b, c). ∗*P* < 0.05, ∗∗∗∗*P* < 0.0001; ^#^*P* < 0.05, ^####^*P* < 0.0001. One-way ANOVA with Tukey post-hoc test (d–g). All data are mean ± SEM with the n number indicated in parentheses.

We next pretreated mice with *i.t.* furosemide (10 nmol) or vehicle 20 min before Compound 6 administration (Fig. 11a). In male ICS mice, Compound 6 induced an anti-allodynic effect, evidenced by significantly restoring PWTs at 40 min (dark green vs. cyan symbols), lasting for about 120 min (Fig. 11b), consistent to the duration observed in the ASI model (Fig. 4b–e). However, the effect of Compound 6 was reduced in mice when pretreated with *i.t.* furosemide (Fig. 11b). In male mice without ICS, *i.t.* furosemide alone significantly decreased PWTs (Fig. 11b and d), consistent with the observation in the control group during performing ASI experiments. Also, further analyses using AUC for PWTs at 0–120 min, confirm that furosemide pretreatment significantly prevented the anti-allodynic effect of Compound 6 in male ICS mice (Fig. 11e).

In females, Compound 6 (3 and 10 mg/kg) attenuated mechanical allodynia, up until 120 min in the *i.t.* vehicle-pretreated ICS mice (Fig. 11c). However, the anti-allodynic effect of Compound 6 was attenuated in mice when pre-treated with *i.t.* furosemide (10 nmol). Further analyses using AUC for PWTs at 0–120 min, indicate that *i.t.* furosemide pretreatment significantly prevented the anti-allodynic effect of Compound 6 (Fig. 11g). In female non-ICS mice, furosemide (10 nmol, *i.t.*) pre-treatment slightly, but insignificantly, reduced PWTs (Fig. 11c and f). Thus, in both male and female mice, intrathecal, but not systemic furosemide, significantly attenuated the anti-allodynic effects of Compound 6.

## Discussion

In this study, we found that α6GABA_A_R-selective PAMs, two PQ compounds, attenuated mechanical allodynia and thermal hyperalgesia in ASI and ICS mouse models of fibromyalgia. This occurred in both sexes, while being more potent in females. When benchmarked against existing fibromyalgia medication, their antinociceptive efficacy was not inferior. After repeated administrations, PQs displayed robust anti-allodynic effects without tolerance. PQ's anti-nociceptive effect was abolished in *Gabra6*-knockout mice, and prevented by intrathecal, but not systemic, blockade of α6GABA_A_Rs. These results suggest that PQs reduce chronic widespread nociceptive responses in both fibromyalgia models, cardinally through spinally expressed α6GABA_A_Rs.

GABA_A_Rs have long been proposed as druggable targets for pain relief [10]. However classical benzodiazepine non-selective GABA_A_R PAMs are unable to relieve pain at non-sedative doses [47]. Here, we revealed an alternative pharmacological approach, using subtype-selective ligands, α6GABA_A_R-selctive PAM PQs. They attenuated nociceptive responses in two fibromyalgia models at doses without significant sedation, motor impairment, or addictive liability [22,24,48]. The ICS and ASI models represent chronic widespread nociplastic pain induced by central sensitization, triggered by cold stress [49] and peripheral muscle acidic-saline injection [50], respectively. The consistent efficacy of PQs across these mechanistically distinct models supports a central modulatory role of PQs in fibromyalgia-like pain.

α6GABA_A_Rs have been identified in the spinal cord of mice [42], rats [17], turtles [18], and humans [19]. We further confirmed the expression of α6GABA_A_Rs in superficial, intermediate and deep laminas of the mouse spinal dorsal horn. The colocalization of the α6 subunit with NeuN infers that α6GABA_A_Rs are expressed in the cell body of secondary sensory neurons in the spinal dorsal horn. Interestingly, there was some co-immunoreactivity of the α6 subunit and IB4, a primary sensory neuronal marker, in the spinal dorsal horn. It remains to be further elucidated using electron microscopy whether the α6 subunit is located pre-synaptically at central terminals of IB4-positive primary sensory neurons, or post-synaptically on spinal secondary sensory neurons innervated by IB4-positive terminals. Nevertheless, as intrathecal furosemide blocked PQ's antinociceptive effects, it strongly supports the spinal cord as the primary action site.

As furosemide also inhibits Na–K–Cl co-transporter 1 (NKCC1) [51] and potassium-chloride co-transporter 2 (KCC2) [52,53], a low dose *i.t.* furosemide was selected which does not affect KCC2, due to having a greater binding affinity to α6GABA_A_Rs [29]. Regarding NKCC1, upregulation was reported at DRG [54] and spinal dorsal horn [55] of animals with neuropathic pain, and NKCC1 inhibition reduced nociceptive responses [56,57]. In contrast, *i.t.* furosemide reduced PWTs in non-ASI and non-ICS mice here. Thus, NKCC1 inhibitory activity of furosemide is unlikely contribute to its reversal of PQs’ antinociceptive effects.

In rodent TG, α6GABA_A_Rs are identified in satellite glial cells [24] and astrocytes [58]. If α6GABA_A_Rs were present in spinal astrocytes, potentiation of α6GABA_A_Rs by PQs would be expected to suppress astrocyte activity, which may induce anti-nociceptive rather than pro-nociceptive effects, given that spinal astrocyte gliopathy was implicated in chronic pain [59,60]. There is currently no clear evidence supporting the presence of α6GABA_A_Rs in microglia [19]. Thus, a microglia-mediated mechanism may not account for the anti-nociceptive effects of PQs.

Furosemide, which is BBB-impermeable [19,41], did not reverse PQs’ antinociceptive effects, and there was no significant α6-immunoreactivity in the DRG. Therefore, the action site of PQs is not cardinally at the DRG of ICR mice. In contrast, α6GABA_A_Rs were reported in DRG neurons of rats recently [19]. This discrepancy may be due to species differences [61] or technical limitations e.g., α6GABA_A_R expression levels in mice may be too low for detection by current available mouse antibodies. Alternatively, this may be due to trafficking of α6GABA_A_Rs away from the DRG soma, toward peripheral and central processes [62,63]. Nevertheless, earlier studies failed to detect *Gabra6* mRNA at the DRG of rats [64], mice [[65], [66], [67]] and humans [68]. Therefore, it is unclear whether the α6GABA_A_R expression observed in rat DRG neurons contributed to the PQ effect on nociceptive signaling. Alternatively, interspecies differences in GABA_A_R-subunit composition and expression patterns may underlie the divergent findings [61]. For example, α6GABA_A_Rs may follow the expression pattern of Gabrd (the δ-subunit of GABA_A_Rs). In rats and mice, *Gabrd* was shown absent or very low at DRG [64,65,69,70], whereas *Gabrd* and Gabrd was abundant in the spinal cord [[71], [72], [73]].

Since existing GABAergic drugs have shown gender difference effects [74,75], it became valuable to determine whether males and females respond similarly to PQ treatment. In neuropathic rats, *i.t.* PQs were reported to induce a female-specific, male-resistant, anti-allodynic effect, peaking ∼3 h after drug administration [19]. This female-specific PQ effect was attributed to an estrogen-dependent restoration of downregulated α6GABA_A_R expression in the female spinal dorsal horn, and later shown in additional studies including neuropathic and reserpine fibromyalgia models [76]. Additionally, it was also found that both activation and organizational actions of gonadal hormones shaped the sexual dimorphism of α6GABA_A_R-mediated antinociception in neuropathic pain [77]. Therefore, gonadal hormone regulation of α6GABA_A_R expression or function may be a conserved mechanism across pain modalities, and compound effects. Here, we found both sexes responsive to PQs, while females were more sensitive, in both ASI and ICS fibromyalgia models. Moreover, pharmacological effects of PQs in mice consistently peaked within 30–40 min across our behavioral studies [[22], [23], [24],^39^,^48^,^78^], including the present study. Female mice displayed more prominent anxiety-like behavior, which is interesting since women reportedly show higher prevalence rates of anxiety disorders [79], and in fibromyalgia, women may have higher rates of psychiatric disorder commodity [80]. Nevertheless, the dimorphic response to PQs in mechanical allodynia at the spinal level, may be attributed to pharmacokinetic differences between sexes, hormonal modulation of α6GABA_A_Rs expression or function [76,77,79], or other biological variables.

Recent evidence has suggested that the cerebellum may play an important role in anxiety [81]. This is interesting since, α6GABA_A_Rs are predominantly expressed in cerebellum GCs [17], as opposed to other brain regions, which express other GABA_A_R subtypes [82], particularly at supraspinal circuitry which traditionally underlie anxiety disorders [83]. Also, α6GABA_A_Rs are implicated in several aspects of mood regulation, including anxiety and stress [84]. In humans, an association between *GABRA6* variants and anxiety disorders has been suggested [85]. However, other studies did not find a direct association with *GABRA6* and anxiety [86,87]. Notably, a recent study which employed conditional knockout mice, by deleting cerebellar GC synaptic transmission using two *Gabra6*-Cre lines, found no significant difference in anxiety-like behavior versus control mice [88]. The authors suggested that anxiety-like behavior may be cerebellar cortex-dependent, without GC-dependent processing. Although compensatory changes cannot be ruled out in the constitutive GC-*Gabra6*-Cre mice, as α6GABA_A_Rs are almost exclusively expressed in GCs [17], this may explain the lack of anxiolytic effect by Compound 6 in our study. Thus, the persistence of anxiety-like behaviors, despite the robust antinociceptive effects of Compound 6 in the ASI fibromyalgia model, suggests that affective disturbances in fibromyalgia may not be solely attributable to chronic pain, and could involve, at least in part, a mechanism distinct from that underlying pain hypersensitivity.

In fibromyalgia patients, sensitization of spinal dorsal horn neurons and increased receptive fields were associated with pain severity [89]. Moreover, they had fewer GABA [13], and greater excitatory transmitters [[90], [91], [92]] in spinal cerebrospinal fluid, than in normal controls. Thus, impaired GABAergic transmission in the spinal dorsal horn may impact wide-spread pain in fibromyalgia. Our finding that *i.t.* furosemide reduced PWTs in control mice suggests α6GABA_A_R-mediated GABAergic tone in the spinal cord provides an anti-nociceptive protection physiologically. Also, we did not observe a basal PWT difference between *Gabra6*-knockout and *Gabra6*-wild-type mice. Interestingly, mice with global knockout of *Gabrad* or *Gabra4* (the α4-subunit of GABA_A_Rs) did not show basal sensitivity changes in response to foot shock [93] or thermal [94] stimulation. These negative findings may reflect possible compensatory change(s) in other GABA_A_R subunits in global knockout mice [95]. In contrast, in pathological conditions where baseline PWTs were markedly reduced, *i.t.* furosemide did not further suppress PWTs, suggesting endogenous GABAergic protection may be insufficient or functionally impaired. However, when PQs were administered, spinal α6GABA_A_R-mediated GABAergic transmission may be potentiated, leading to enhanced inhibitory control over nociceptive input from primary afferents. This may explain how PQs exert their antinociceptive effects in fibromyalgia models.

Gabapentinoids are the first line choice clinically for managing chronic widespread pain in fibromyalgia patients [30]. They bind to α_2_δ receptors of calcium channels [96], and in chronic pain pathology, weaken glutamate release at presynaptic terminals in the spinal cord [97]. In-spite of their therapeutic usage, they are dose-limited by unwanted adverse effects [98], such as peripheral edema [99], sedation and cognitive impairment [100]. Thus, an unmet medical need exists for safe and effective fibromyalgia treatments. Here, PQs effectively attenuated nociceptive responses in two fibromyalgia models with the efficacy comparable to gabapentin. The PQ DK-I-56-1, displayed a similar action duration as gabapentin. Also, combined DK-I-56-1 and gabapentin treatment at low doses produced an additive anti-allodynic effect. Thus, clinically, DK-I-56–1 may serve as a potential novel treatment strategy for fibromyalgia as an add-on therapy with low dosed gabapentinoids to avoid side effects [100].

In previous studies, we demonstrated that PQs, including DK-I-56-1, exhibited a favorable safety pharmacology profile. This was characterized by the absence of off-target activity among 46 screening targets, including arrhythmogenic MinK channels, and lack of hepatic or renal toxicity [22]. Importantly, preclinical toxicity studies demonstrated that DK-I-56-1 did not induce benzodiazepine-like side effects, such as sedation, motor impairment, or addictive potential [22,24,48]. Taken together, these findings support DK-I-56-1 as a druggable candidate for development as a novel monotherapy for fibromyalgia.

Here, we identified α6GABA_A_Rs in the spinal dorsal horn as a potential novel therapeutic target for pain relief in fibromyalgia patients. PQ compounds, which act as α6GABA_A_R-selective PAMs in the spinal dorsal horn, produced antinociceptive effects in two mechanistically distinct mouse models of fibromyalgia without detectable tolerance, and were effective in both sexes with greater sensitivity in females. Among these, DK-I-56-1 demonstrated allodynic efficacy and duration comparable to gabapentin, and showed additive effects when co-administered with gabapentin. Given that current treatments for fibromyalgia are limited by dose-dependent adverse effects and modest efficacy, α6GABA_A_R-selective PAMs, such as DK-I-56-1, warrant further investigation as candidate therapeutic approaches, either as monotherapy or as adjuncts to gabapentinoids.

## Data availability

Data will be made available on request.

## Author contributions

**Myles Sant-Cassia**: Conceptualization, Investigation, Formal analysis, Methodology, Visualization, Writing—Original Draft. **Cheng-Han Lee**: Validation, Methodology, Writing—Review & Editing. **V. V. N. Phani Babu Tiruveedhula**: Resources, Writing – review & editing. **James Cook**: Resources, Writing – Review & Editing. **Chih-Cheng Chen**: Supervision, Validation, Writing—Review & Editing. **Lih-Chu Chiou**: Conceptualization, Methodology, Project administration, Supervision, Validation, Visualization, Resources, Funding Acquisition, and Writing— Review & Editing.

## Declaration of competing interest

The authors declare that they have no known competing financial interests or personal relationships that could have appeared to influence the work reported in this paper.
