# Supplementary material for: α6GABAA receptor-selective positive allosteric modulator as a novel therapy for fibromyalgia: A proof-of-concept study in mice modeling chronic widespread musculoskeletal pain
Source: Neurotherapeutics. 2026 Jul 16;23(4):e00956. doi: 10.1016/j.neurot.2026.e00956 (PMC13382795; doi:10.1016/j.neurot.2026.e00956)
Supplement: Multimedia component 1 [file mmc1.pdf]

a

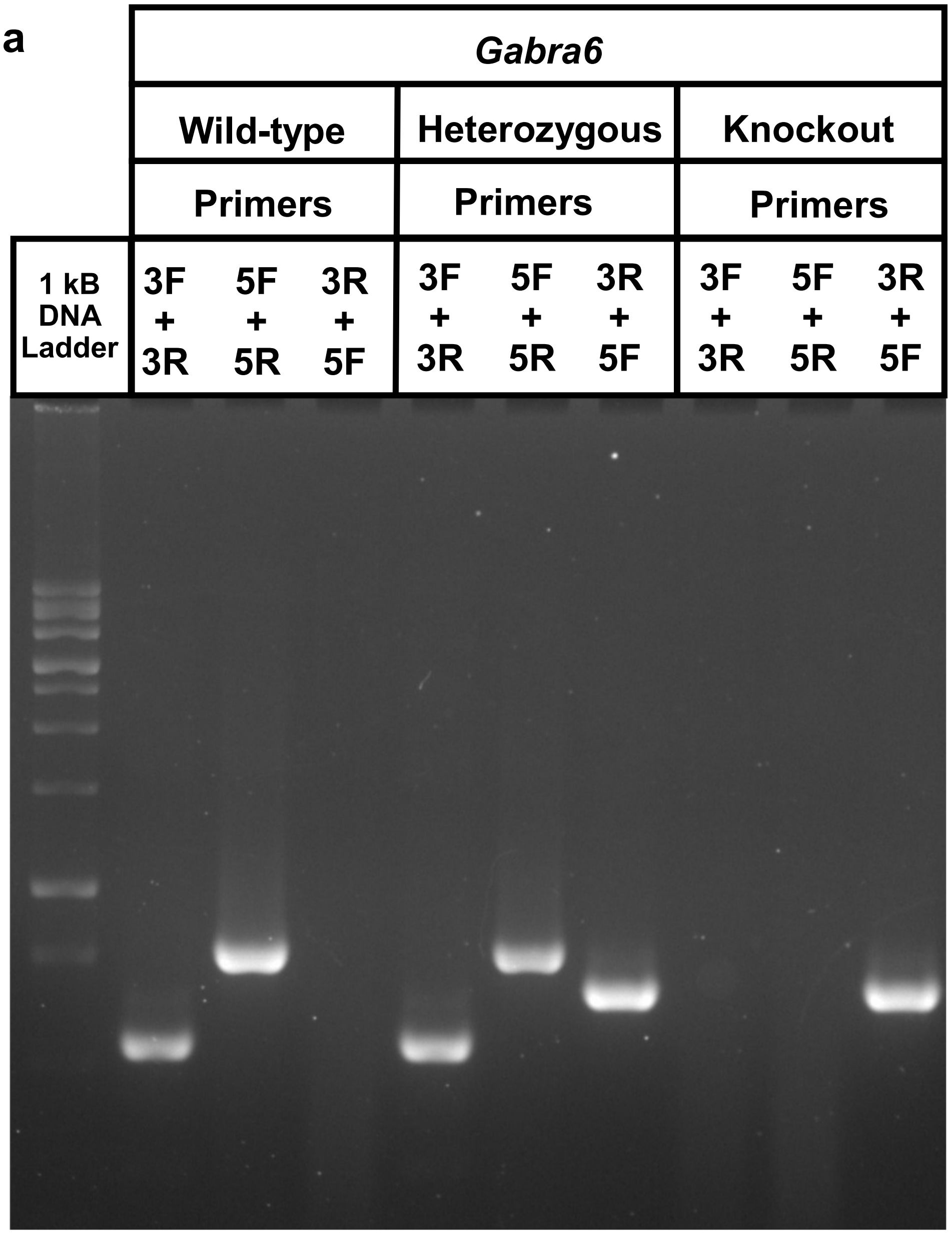

Figure S1. Representative genotyping result for *Gabra6*-knockout mice. a: PCR genotype of *Gabra6* wild-type (*Gabra6* $^{+/+}$), heterozygous (*Gabra6* $^{+/-}$), and knockout (*Gabra6* $^{-/-}$) mice. A total of four primers were used: *Gabra6*-3 forward (3F), *Gabra6*-3 reverse (3R), *Gabra6*-5 forward (5F), and *Gabra6*-5 reverse (5R). For the PCR, mouse samples were tested with three primer combinations: 3F + 3R, 5F + 5R, or 3R + 5F. After gel electrophoresis (100 V, 500 mA), each *Gabra6* genotype showed a distinct profile, representative by a positive signal (white band) or negative signal (no white band).

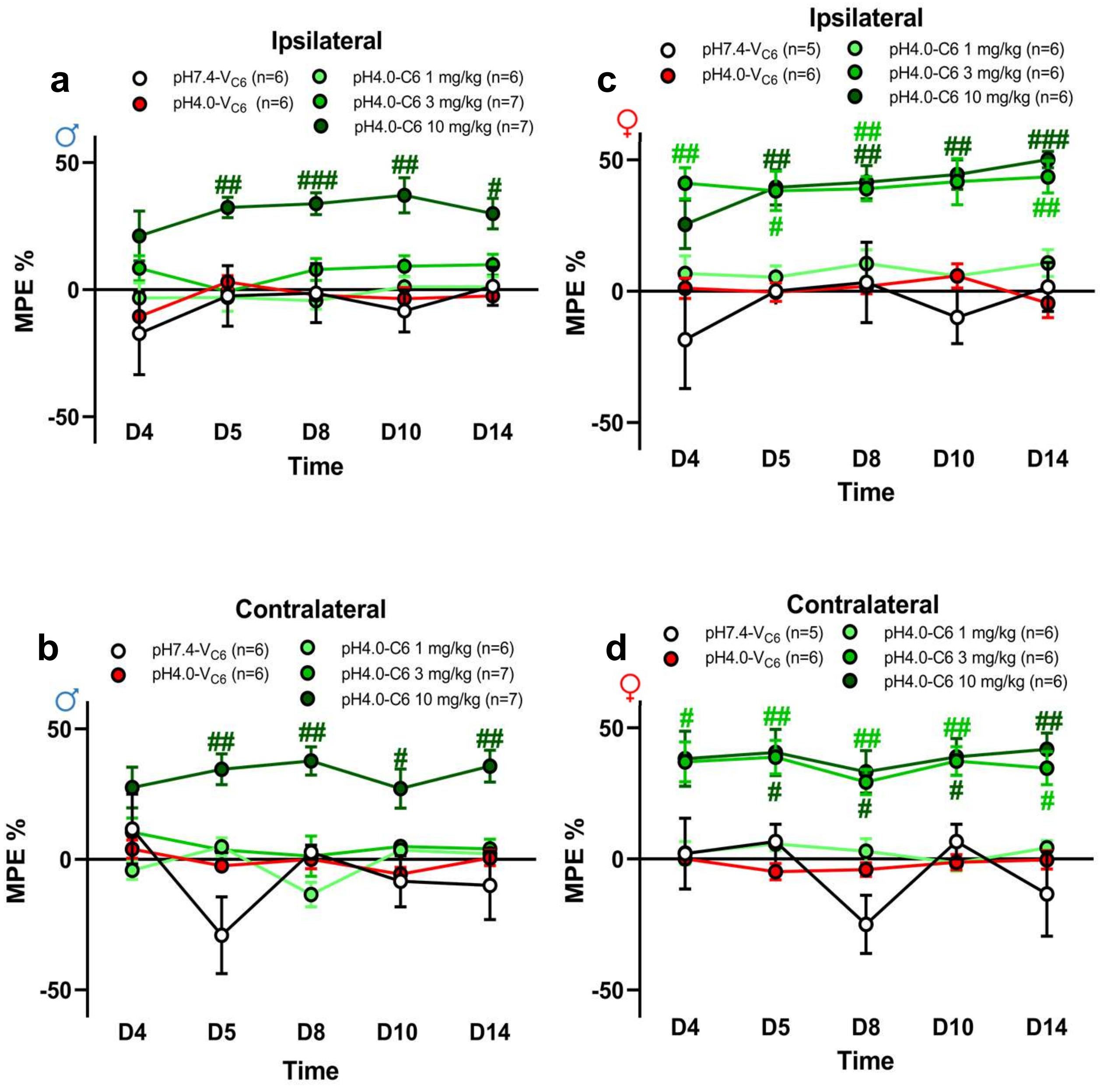

Figure S2. Evaluating anti-allodynic tolerance of Compound 6 in dual acidic-saline-injected (ASI) ICR mice. The time course of the maximum possible anti-allodynic effect (MPE %) of Compound 6 (1, 3, 10 mg/kg, *i.p.*) in ipsilateral (a, b) and contralateral (c, d) sides of male (a, b) and female (c, d) mice. MPE was calculated by the paw withdrawal threshold (PWT) obtained in Figure 1b-e, days 4-14, as described in Materials and Methods. $^{\#}P<0.05$, $^{\#\#}P<0.01$, $^{\#\#\#}P<0.001$, *vs.* pH4.0-V_{C6}. One-way repeated measures ANOVA with Tukey post-hoc test. Data are mean ± SEM with the n number indicated in parentheses.

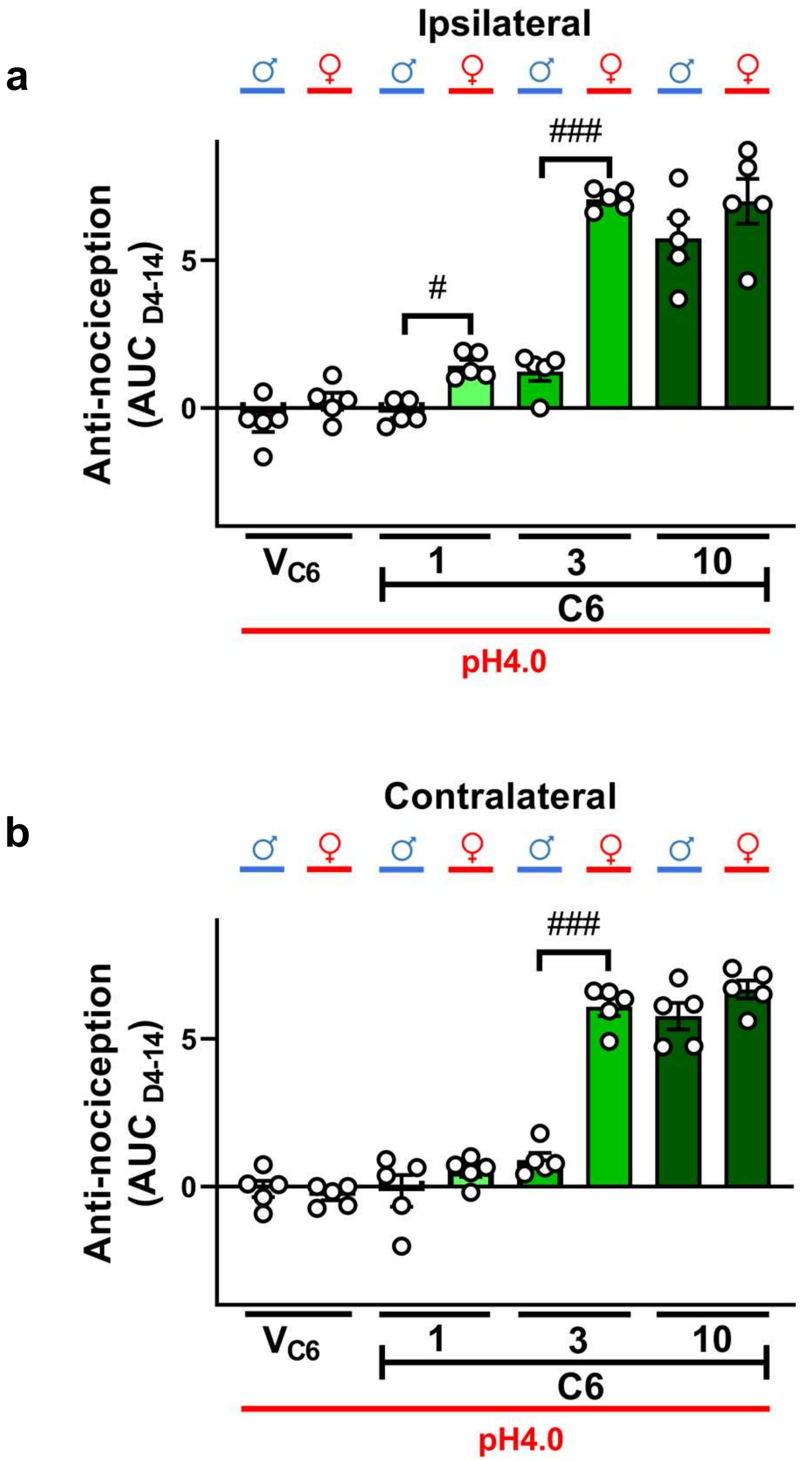

Figure S3. Evaluating the anti-allodynic effect of Compound 6 in male and female dual acidic-saline-injected (ASI) ICR mice. a, b**:** Anti-nociception effects of Compound 6 in male and female mice with ASI were quantified as the cumulative net PWT AUC across days 4-14, referenced to the daily baseline PWT in each mouse. Data obtained from Figure 1b-e. #P<0.05, ###P<0.001. One-way ANOVA with Tukey post-hoc test.

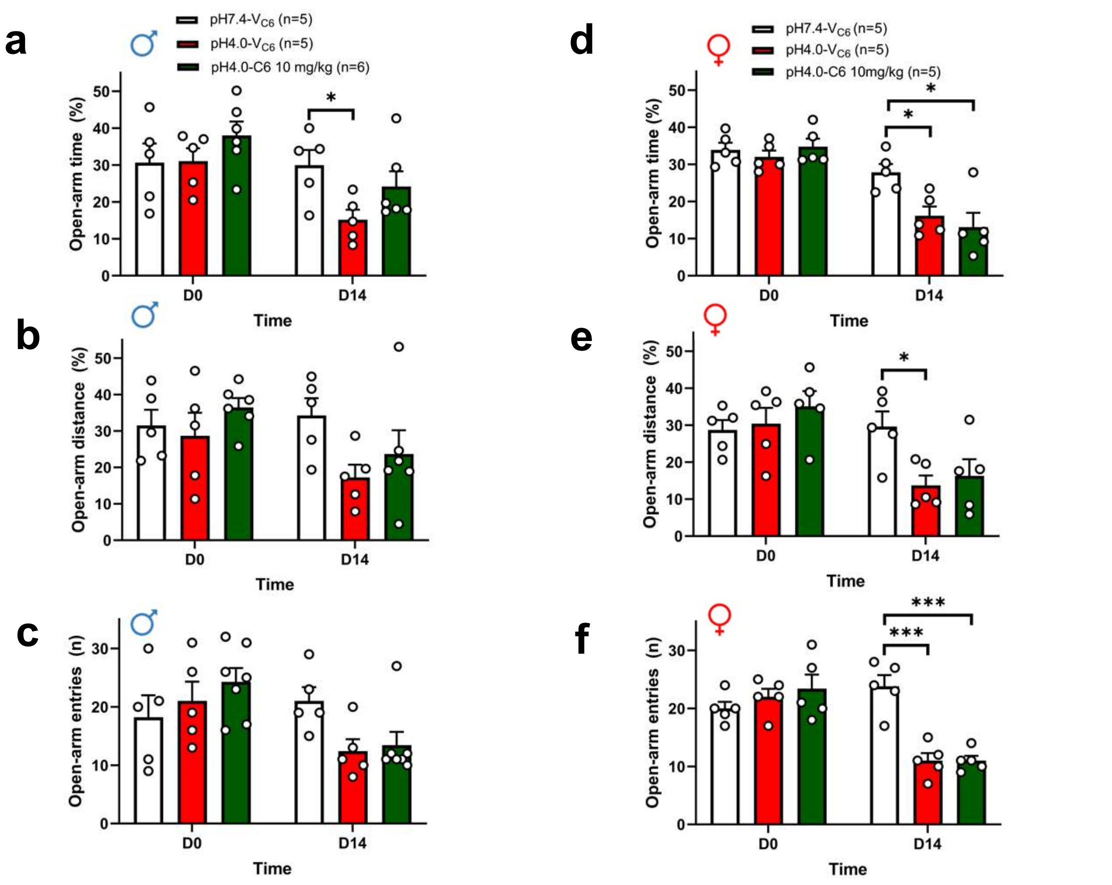

Figure S4. Evaluating anxiety-like behaviors in the dual acidic-saline injection (ASI) model. ASI was performed in mice with the same protocol as in Figure 1. Before and 14 days after ASI, anxiety-like behaviors were evaluated by the elevated plus maze test in male (a-c) and female (d-f) mice. Compound 6 (10 mg/kg) or vehicle was *i.p.* administered 20 minutes before the test. $^{*}P<0.05$, $^{***}P<0.001$, *vs.* pH7.4V$_{C6}$. One-way ANOVA with Tukey post hoc test. Data are mean ± SEM with the n number indicated in parentheses.

a

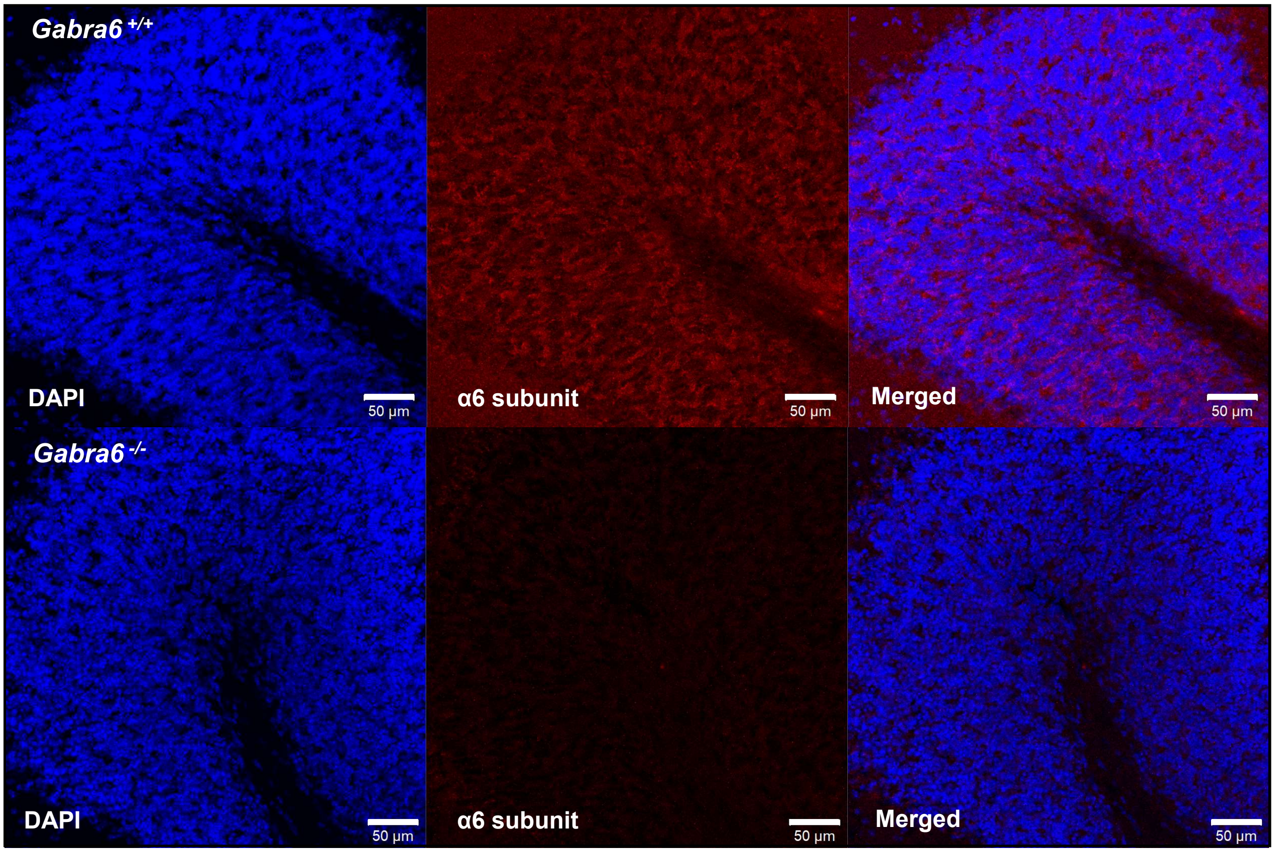

b

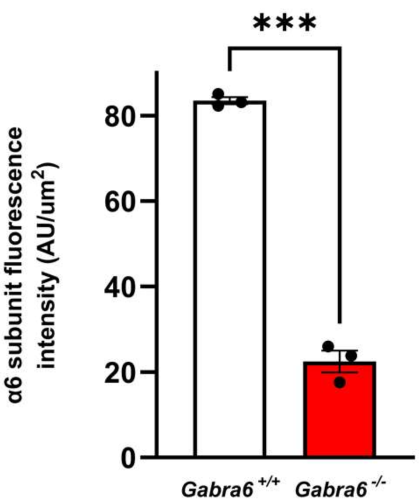

Figure S5. Immunofluorescent staining of the α6 subunit representing the α6GABA$_A$R at the cerebellum. a: Immunofluorescence staining of the α6 subunit (red), representing the α6GABA$_A$R, and DAPI (blue) in 30 μm-thick cerebellar sections prepared from male *Gabra6* $^{+/+}$ and *Gabra6* $^{-/-}$ mice. Note that the α6 subunit (red) was densely stained in the cells, stained by DAPI (blue), in the cerebellar granule layer in the *Gabra6* $^{+/+}$, but was absent in *Gabra6* $^{-/-}$ group. The same result was reproduced in 3 mice of each group. (b) Statistical analysis of the α6 subunit fluorescence intensity was performed using the student t-test. Scale bars represent 50 μm.

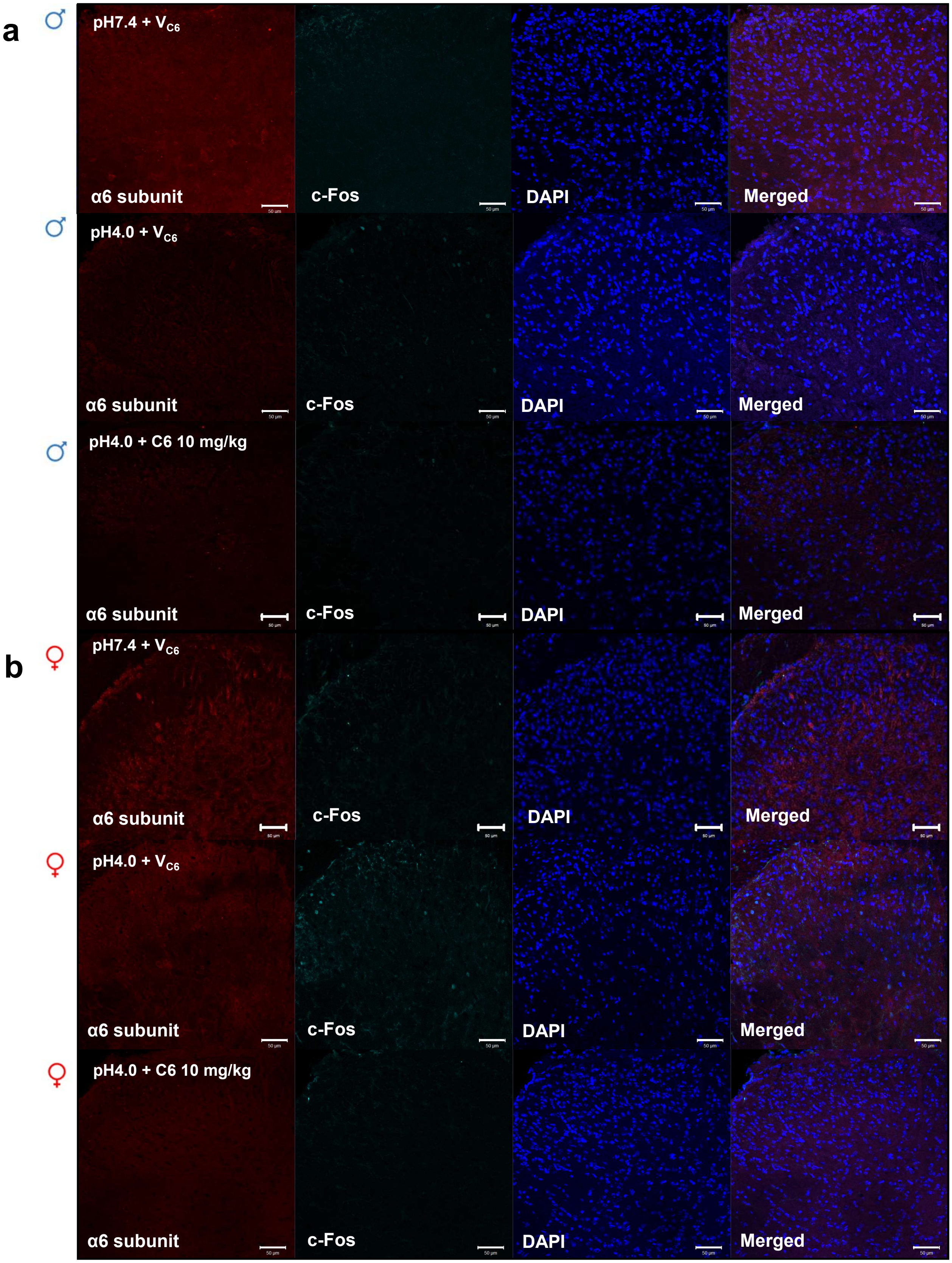

Figure S6. Immunofluorescent staining of c-Fos at the spinal cord. a, b: Immunofluorescence staining of the α6 subunit (red), representing the α6GABA$_A$R, c-Fos (cyan), and DAPI (blue) in the spinal dorsal horn in male (a) and female (b) mice in the ASI model. Spinal cord sections were prepared in mice 90 minutes after the repeated von-Frey test, and 4 days after ASI. Compound 6 (10 mg/kg) or vehicle was *i.p.* administered 20 minutes before the test as described in Materials and Methods. Note that c-Fos was reduced in mice with Compound 6 treatment. The same result was reproduced in 3 mice of each group. Scale bars represent 50 μm.

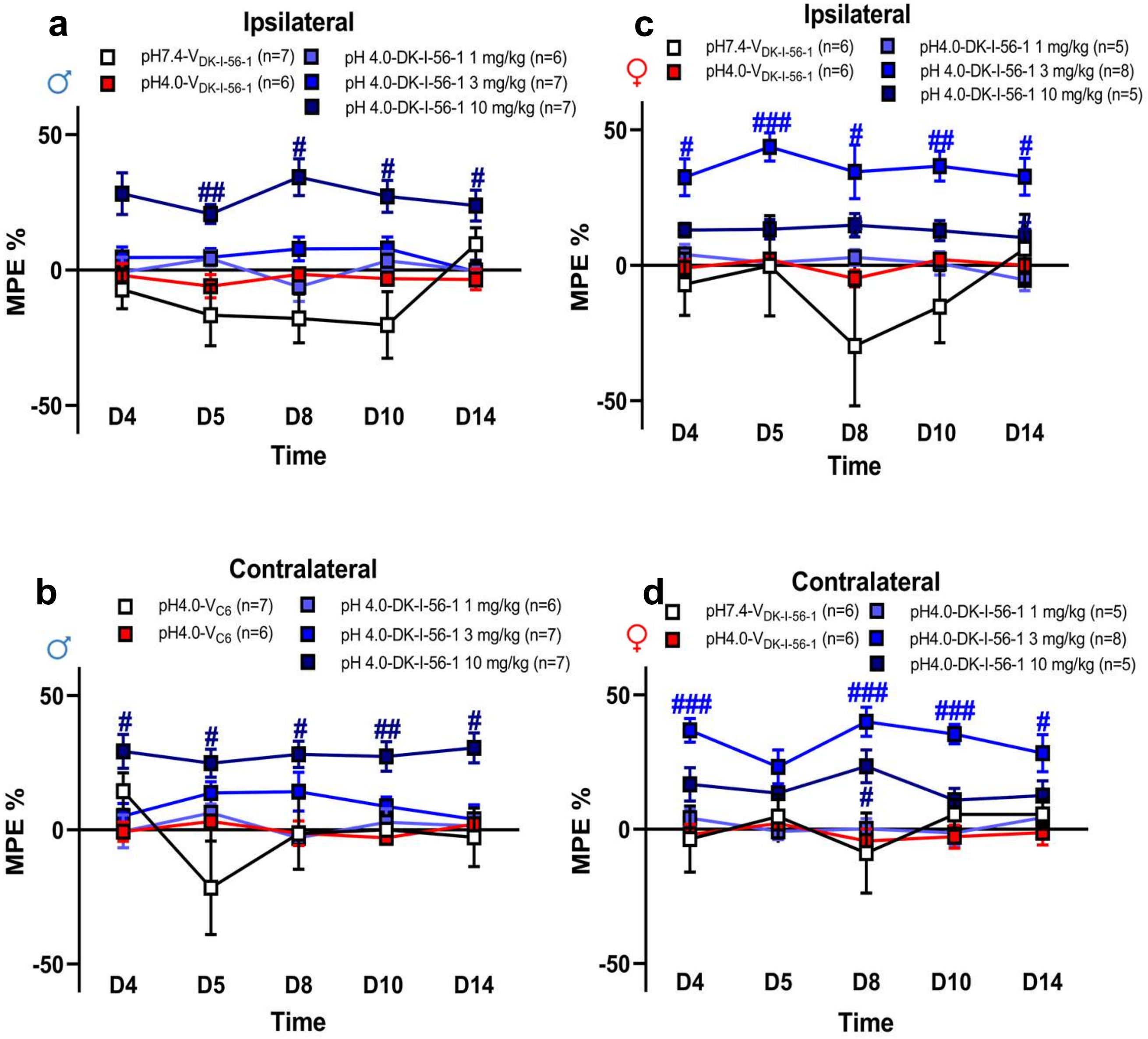

Figure S7. Evaluating anti-allodynic tolerance of DK-I-56-1 in dual acidic-saline-injected (ASI) ICR mice. The time course of the maximum possible anti-allodynic effect (MPE %) of DK-56-1 (1, 3, 10 mg/kg, *i.p.*) in ipsilateral (a, b) and contralateral (c, d) sides of male (a, b) and female (c, d) mice. MPE was calculated by the paw withdrawal threshold (PWT) obtained in Figure 5b-e, days 4-14, as described in Materials and Methods. $^{\#}P<0.05$, $^{\#\#\#}P<0.001$, *vs.* pH4.0-V_{C6}. One-way repeated measures ANOVA. Data are mean ± SEM with the n number indicated in parentheses.

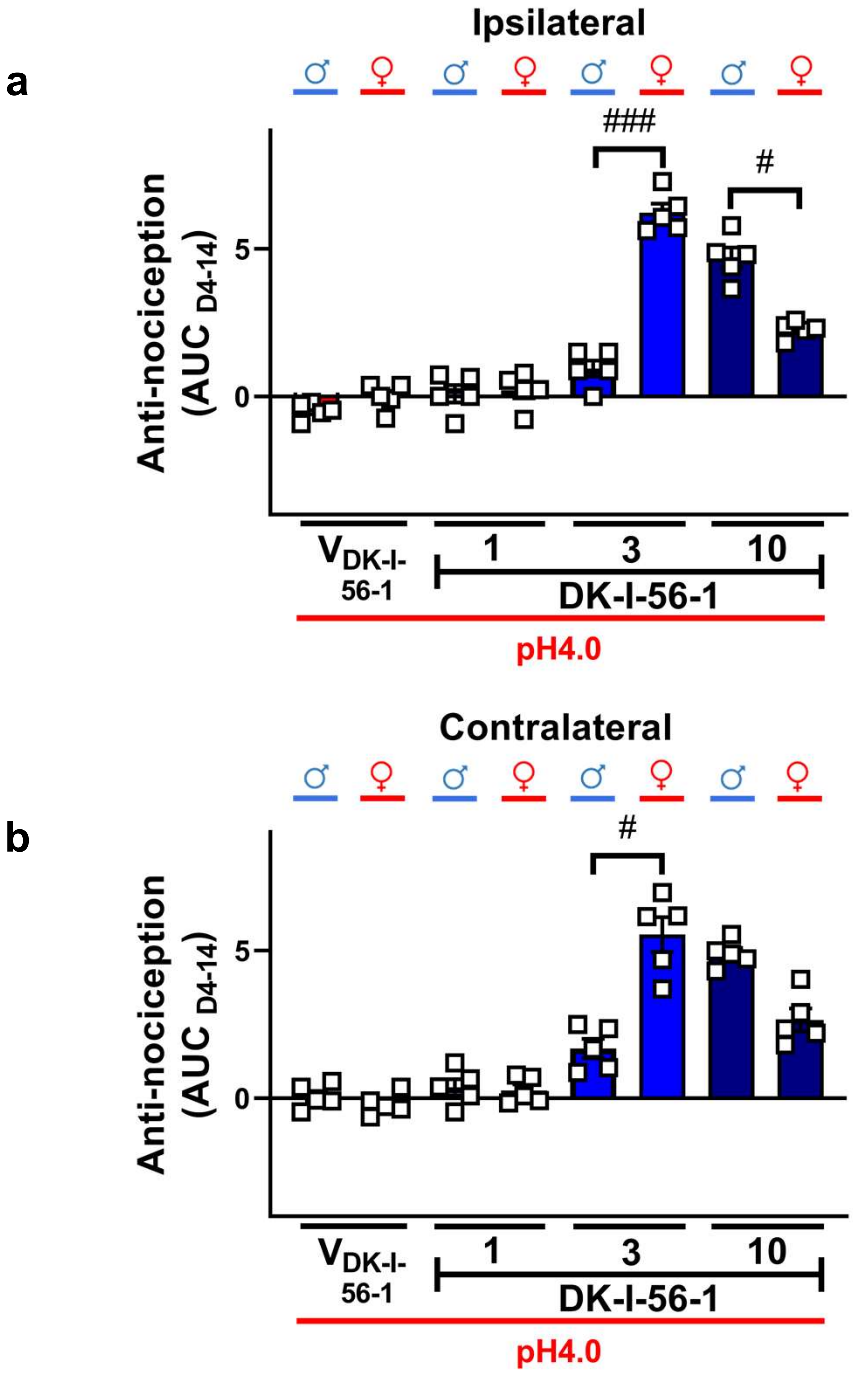

Figure S8. Evaluating the anti-allodynic effect of DK-I-56-1 in male and female dual acidic-saline-injected (ASI) ICR mice. a, b**:** Anti-nociception effects of DK-I-56-1 in male and female mice with ASI were quantified as the cumulative net PWT AUC across days 4-14, referenced to the daily baseline PWT in each mouse. Data obtained from Figure 6b-e. $^{\#}P<0.05$, $^{\#\#\#}P<0.001$. One-way ANOVA with Tukey post-hoc test.

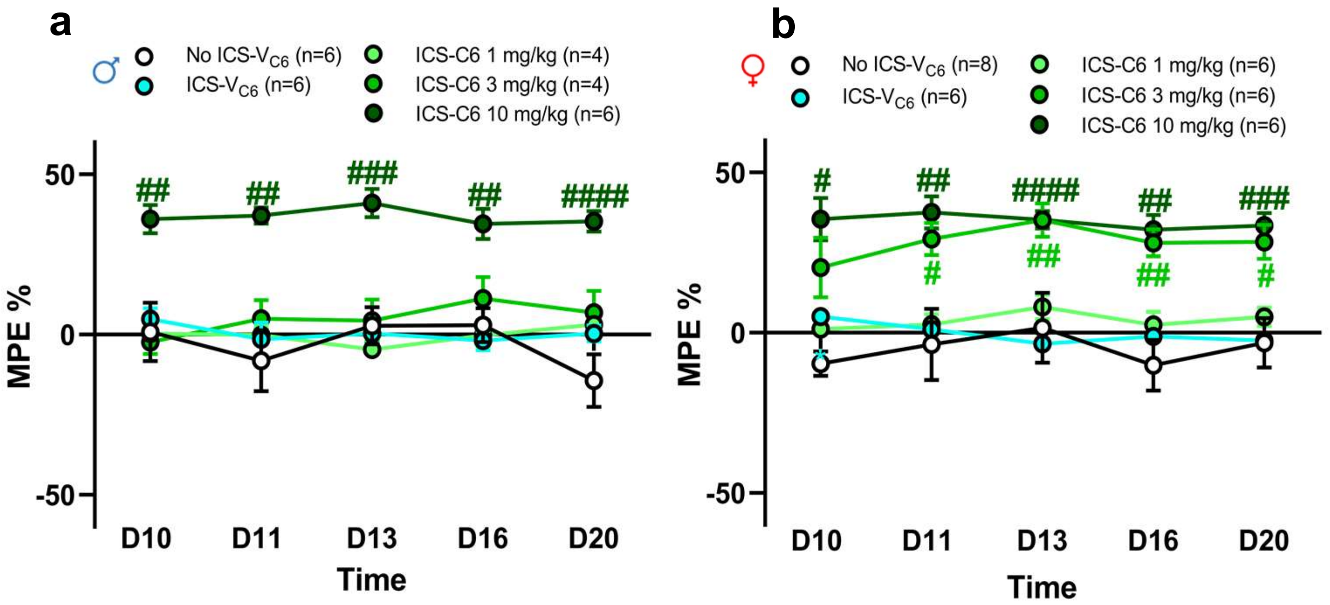

Figure S9. Evaluating anti-allodynic tolerance of Compound 6 in intermittent cold stress (ICS) ICR mice. The time course of the maximum possible anti-allodynic effect (MPE %) of Compound 6 (1, 3, 10 mg/kg, *i.p.*) in male (a) and female (b) mice. MPE was calculated by the paw withdrawal threshold (PWT) obtained in Figure 8b, c, days 10-20, as described in Materials and Methods. $^{\#}P<0.05$, $^{\#\#}P<0.01$, $^{\#\#\#}P<0.001$, *vs.* pH4.0-V$_{C6}$. $^{\#}P<0.05$, $^{\#\#}P<0.01$, $^{\#\#\#}P<0.001$, $^{\#\#\#\#}P<0.0001$, *vs.* ICS-V$_{C6}$. One-way repeated measures ANOVA. Data are mean ± SEM with the n number indicated in parentheses.

a

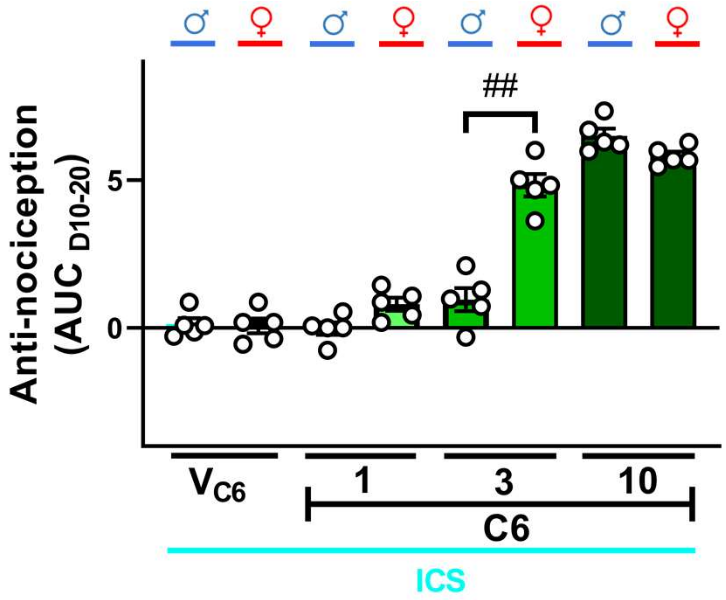

Figure S10. Evaluating the anti-nociception effect of Compound 6 in male and female intermittent cold stress (ICS) ICR mice. a**:** Anti-nociception effects of Compound 6 in male and female mice with ICS were quantified as the cumulative net PWT AUC across days 10-20, referenced to the daily baseline PWT in each mouse. Data obtained from Figure 9b, c. $^{\#\#}$P<0.01. One-way ANOVA with Tukey post-hoc test.

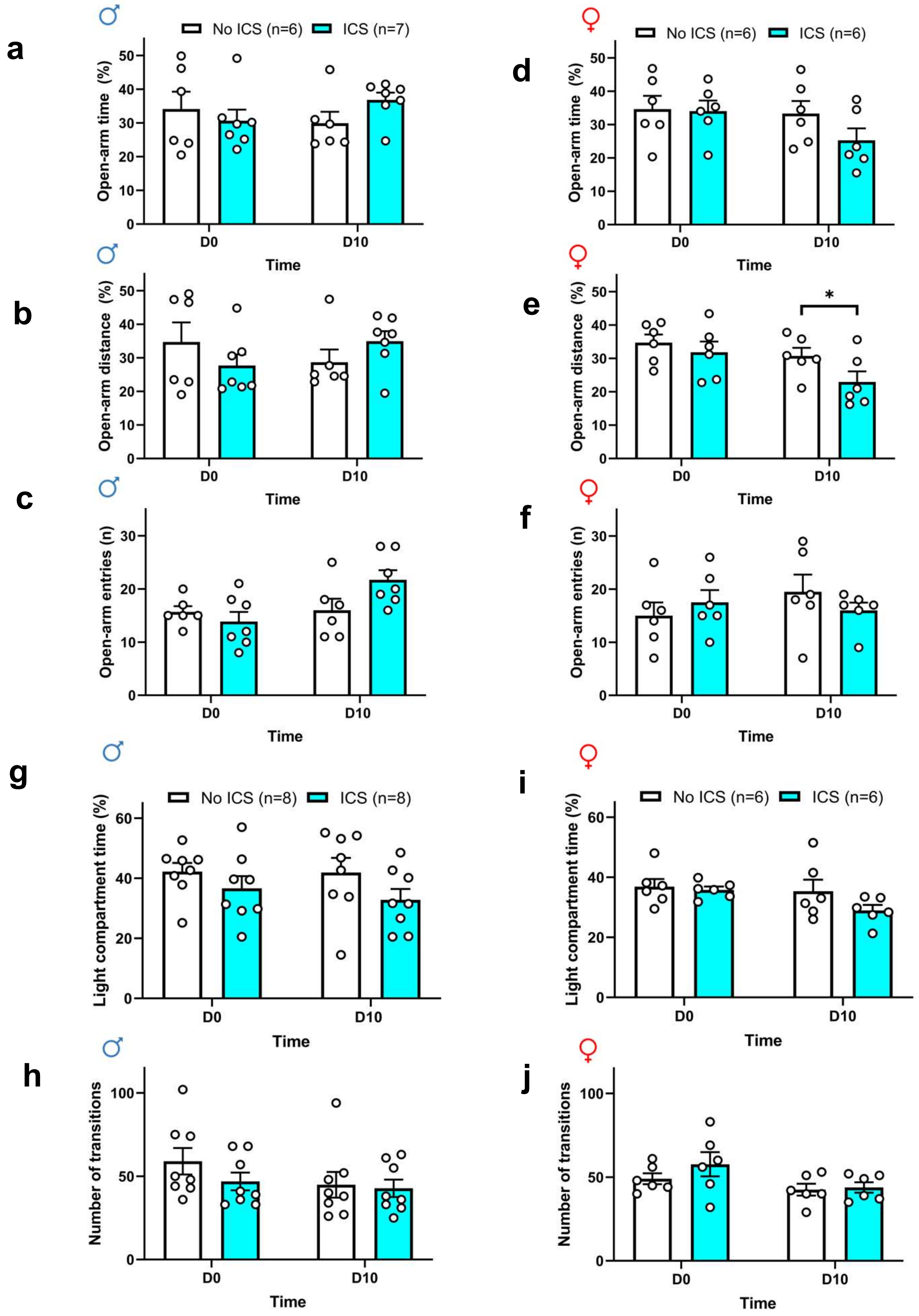

Figure S11. Evaluating anxiety-like behaviors in the intermittent cold stress (ICS) model. ICS was performed in mice with the same protocol as in Figure 8. Before and at day 10 after ICS, anxiety-like behaviors were evaluated by the elevated plus maze test (a-f) or light/dark box test (g-j) in male (a, b, c, g, h, i) and female (d, e, f, i, j) mice. $^{*}P$<0.05, *vs.* no ICS. Mann–Whitney test. Data are mean ± SEM with the n number indicated in parentheses.
